# OmpA signal peptide leads to heterogenous secretion of *B. subtilis* chitosanase enzyme from *E. coli* expression system

**DOI:** 10.1186/s40064-016-2893-y

**Published:** 2016-07-28

**Authors:** Phornsiri Pechsrichuang, Chomphunuch Songsiriritthigul, Dietmar Haltrich, Sittiruk Roytrakul, Peenida Namvijtr, Napolean Bonaparte, Montarop Yamabhai

**Affiliations:** 1 Molecular Biotechnology Laboratory, School of Biotechnology, Institute of Agricultural Technology, Suranaree University of Technology (SUT), 111 University Avenue, Meung District, Nakhon Ratchasima, 30000 Thailand; 2 Synchrotron Light Research Institute (Public Organization), Nakhon Ratchasima, Thailand; 3 Food Biotechnology Laboratory, BOKU - University of National Resources and Life Sciences, Vienna, Austria; 4 Genome Institute, National Center for Genetic Engineering and Biotechnology, Pathumthani, Thailand

**Keywords:** Secretion, Recombinant, *E. coli*, Expression, Signal peptide, OmpA, *Bacillus*, Chitosanase

## Abstract

The production of secreted recombinant proteins from *E. coli* is pivotal to the biotechnological industry because it reduces the cost of downstream processing. Proteins destined for secretion contain an N-terminal signal peptide that is cleaved by secretion machinery in the plasma membrane. The resulting protein is released in an active mature form. In this study, *Bacillus subtilis* chitosanase (Csn) was used as a model protein to compare the effect of two signal peptides on the secretion of heterologous recombinant protein. The results showed that the *E. coli* secretion machinery could recognize both native bacillus and *E. coli* signal peptides. However, only the native bacillus signal peptide could generate the same N-terminal sequence as in the wild type bacteria. When the recombinant Csn constructs contained the *E. coli* OmpA signal peptide, the secreted enzymes were heterogeneous, comprising a mixed population of secreted enzymes with different N-terminal sequences. Nevertheless, the *E. coli* OmpA signal peptide was found to be more efficient for high expression and secretion of bacillus Csn. These findings may be used to help engineer other recombinant proteins for secretory production in *E. coli*.

**Electronic supplementary material:**

The online version of this article (doi:10.1186/s40064-016-2893-y) contains supplementary material, which is available to authorized users.

## Background

The production of secreted recombinant proteins from *E. coli* is pivotal to the biotechnological industry because it reduces the cost of downstream processing associated with non-secreted proteins (Mergulhao et al. 2005). Secreted proteins are usually properly folded and more stable than cytosolic protein because of lower protease levels in the periplasm or culture medium. Proteins destined for secretion carry an N-terminal signal peptide that is cleaved in the plasma membrane by different mechanisms. The resulting protein is released in an active mature form (Mergulhao et al. 2005). *E. coli* is the most commonly used cell factory for the expression and secretion of recombinant enzymes and other biologically active proteins (Mergulhao et al. 2005).

**Fig. 1:**
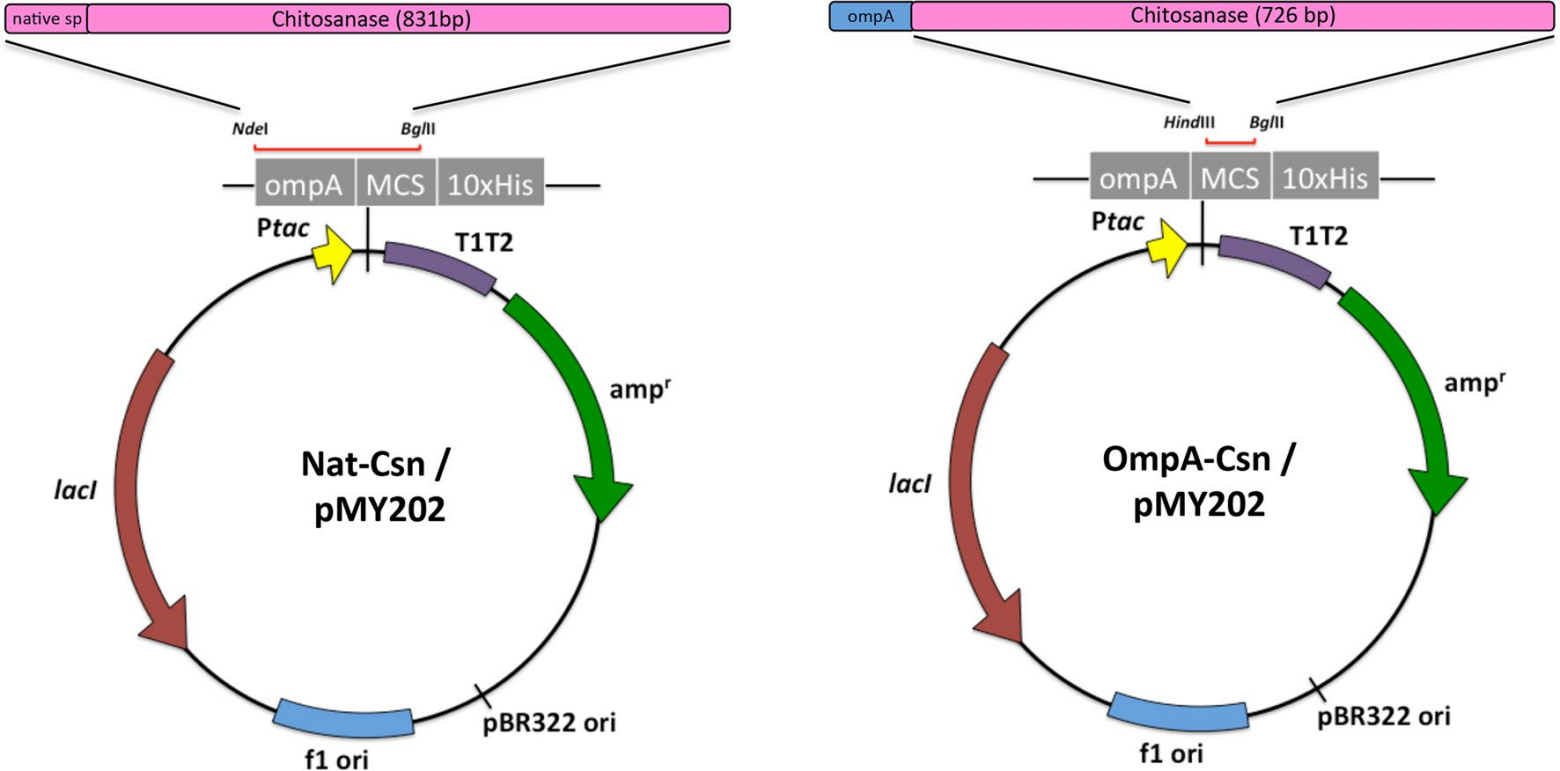
Map of constructs used in this study. Nat-Csn (construct on *left*) was used for secretory expression of Csn via its native bacillus signal peptide, while OmpA-Csn (construct on *right*), comprised of the mature enzyme fused with the *E. coli* OmpA signal peptide that is a component of the pMY202 vector

Since secreted recombinant proteins are fused with an *E. coli* signal peptide, the cleavage site is often unknown and hard to predict because it does not have to be the same as for the natural protein. This trivial issue can have a significant effect on biological activity and protein expression. Despite the importance of this aspect of protein engineering for secretion, there have been only a few reports on the effect of signal peptides on protein expression and subsequent processing in *E. coli*. The aim of this study was to investigate the effect of two different signal peptides on recombinant protein expression and N-terminal processing using chitosanase (Csn) from *Bacillus subtilis* as a model protein. This enzyme is biotechnologically important because it converts chitosan, a recalcitrant waste product from the seafood industry, into value-added chito-oligosaccharides (CHOS), which have been shown to have excellent health and agricultural benefits (Pechsrichuang et al. 2013; Zhou et al. 2015). It is a relatively small, 28-kDa extracellular enzyme that is secreted from *Bacillus subtilis,* a Gram-positive bacteria (Pechsrichuang et al. 2013). To analyze the effect of different signal peptides, a gene encoding mature Csn together with its native signal peptide and a gene encoding recombinant Csn with its native signal peptide replaced with OmpA, a signal peptide from the Gram-negative bacteria *E. coli*, were cloned into a P*tac*-based expression vector and over-expressed in *E. coli* TOP10 cells (Fig. 1). Protein expression levels and enzymatic activity at different time points, in various compartments, and the sequence of the N-terminus of the secreted proteins were determined.

In this article, we show that the type of signal peptide can affect both the structure of the N-terminus and the expression level of recombinant proteins that are secreted from an *E. coli* expression system.

## Results

### Cloning and secretion of recombinant Csn containing two different signal peptides on agar plates

The *tac* promoter was used to control the expression of the two recombinant Csn forms, and protein over-expression was induced with IPTG. The recombinant enzymes were fused with 10× histidine tags at the C-termini to facilitate one-step affinity purification using IMAC, which allowed for accurate determination of production yield and the sequence of N-terminal amino acids.

For the Nat-Csn construct, the native *Bacillus* signal peptide (35 amino acids) was retained by cloning the entire *B. subtilis* Csn gene into the pMY202 vector, which had been previously digested with *Nde*I and *Bgl*II. The PCR products (~831 bp) were digested with *Nde*I and *Bgl*II and cloned into corresponding restriction sites on the pMY202 plasmid (Fig. 1, left panel). For the OmpA-Csn construct, the native signal peptide of *B. subtilis* Csn was replaced with that of the *E. coli* OmpA signal peptide. The PCR products (~726 bp) were digested with *Hind*III and *Bam*HI and cloned into corresponding restriction sites on the pMY202 plasmid, which contained the OmpA gene. This resulted in the fusion of the *E. coli* OmpA signal peptide with the mature enzyme (Fig. 1, right panel). The DNA sequences of the two constructs were confirmed by automated DNA sequencing (Macrogen, Korea). The theoretical molecular mass of the Nat-Csn and OmpA-Csn constructs were 33.23 and 31.26 kDa, respectively.

**Fig. 2:**
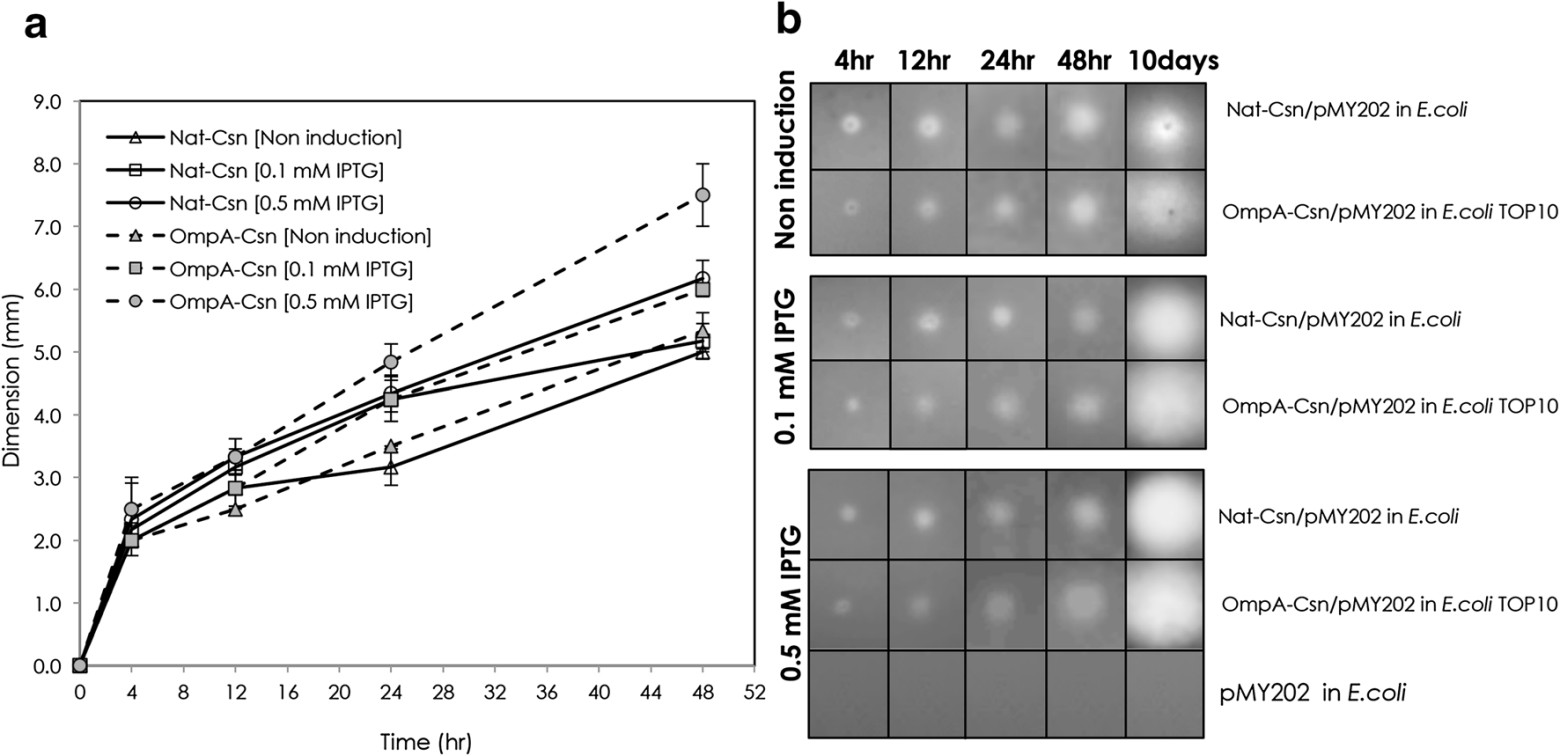
Secretion of recombinant Csn. Mean clear zones diameters (from three colonies of *E. coli* expressing recombinant Nat-Csn and OmpA-Csn) at 0, 4, 12, 24, and 48 h are shown in the panel **a** along with standard error. Representative clear zones from different conditions at 4, 12, 24, 48, and 10 days are shown in the panel **b**. No clear zone was detected around colonies of *E. coli* expressing empty vector (*bottom row*). Enlarged picture of the clear zones can be found in Additional file 1: S6

To compare the effect of the signal peptide on Csn secretion on agar plates, a single colony of *E. coli* TOP10 harboring pMY202 containing either OmpA-Csn or Nat-Csn was grown on LB-Amp agar containing 0.1 % low MW chitosan, and 0, 0.1 or 0.5 mM IPTG, and incubated at 37 °C. Clearing zones from different conditions were measured at different time points and plotted as shown in Fig. 2, left panel. Representative clearing zones are shown on the right panel. While no clearing zones were observed in *E. coli* expressing empty vector, the size of clearing zones of *E. coli* expressing OmpA-Csn were significantly larger than those from Nat-Csn-expressing *E. coli* after 48 h. These results suggest that homologous OmpA is more efficient than the native signal peptide at directing the secretion of heterologous enzyme in *E. coli*. Moreover, these results indicated that over-expression of p*tac* promoter could be induced by increasing concentration of IPTG. The observation of clear zones in the absence of IPTG was an indication that the promoter was leaky.

### Effect of signal peptides on yield and secretion efficiency of recombinant Csn containing two different signal peptides in an *E. coli* expression system

To further evaluate the effect of the signal peptides on the expression and secretion of Csn from *E. coli*, the recombinant proteins were collected from three different compartments (cytoplasm, periplasmic space, and culture broth) and their enzyme activities were analyzed at different time points.

The total enzyme activity in the compartments and the secretion efficiency at different time points are reported in Table 1. These results indicate that the yield of the construct containing the *E. coli* signal peptide was approximately twofold to tenfold higher than the yield of constructs with the native signal peptide, but was dependent on induction time and compartment. At 20 h after induction, both total yield and secretion efficiency of constructs with the OmpA signal peptide were approximately twofold higher than those with the native signal peptide. Moreover, the enzyme activities in the periplasm and culture broth were both approximately fivefold higher when OmpA was used as the signal peptide. At 4 h after induction, when periplasmic leakage should be insignificant (Albiniak et al. 2013; Rinas and Hoffmann 2004), the use of the OmpA signal peptide resulted in as much as a tenfold higher Csn activity in the periplasm. The differences in yield and secretion efficiency were not observed at time 0, when the gene expression was not induced by IPTG. However, leaky expression from the *tac* promoter could be observed as shown in an assay on agar plates. Under these conditions, the secretion efficiency of constructs containing either the *E. coli* or the native signal peptide was equally high.

**Table 1:** Enzyme activity and secretion efficiency

	Induction time (h)	Enzymatic activity (total units)^a^		Fold change^b^
		Native Sp	OmpA Sp	
Broth	0	85.5 ± 2.00	98.0 ± 2.92	1.15
	4	115 ± 1.5	244 ± 5.1	2.12
	20	265 ± 2.0	1386 ± 2.6	5.23
Periplasm	0	0.93 ± 0.02	0.99 ± 0.01	1.06
	4	2.03 ± 0.01	22.2 ± 0.20	10.9
	20	4.90 ± 0.09	27.6 ± 0.69	5.63
Cytosol	0	21.3 ± 0.02	20.5 ± 0.35	0.96
	4	388 ± 2.6	561 ± 12.8	1.45
	20	528 ± 5.1	564 ± 0	1.07
Total	0	108 ± 2.0	119 ± 3.3	1.10
	4	505 ± 1.2	828 ± 7.5	1.64
	20	798 ± 3.0	1978 ± 3.2	2.48
Secretion	0	79.3 ± 0.39	82.0 ± 0.21	1.03
Efficiency	4	22.7 ± 0.34	29.5 ± 0.88	1.30
(%)^c^	20	33.2 ± 0.38	70.1 ± 0.01	2.11

Expression and secretion of *B. subtilis* Csn into different compartments of the *E. coli* host harboring Nat-Csn/pMY202 or OmpA-Csn/pMY202, which contain native (Native Sp) or OmpA (OmpA Sp) signal peptides

^a^Total enzyme activity from 50 mL culture. The experiments were done in duplicate and the average values with standard deviations are reported. The enzyme was induced for over expression with 0.5 mM IPTG

^b^Fold change indicates the relative values of the yields or secretion efficiencies of constructs with different signal peptides from different compartments (OmpA Sp divided by native Sp)

^c^Secretion efficiency was calculated from the percentage of the enzyme activity from culture broth plus periplasmic space divided by the total enzyme activities from all three compartments

**Fig. 3:**
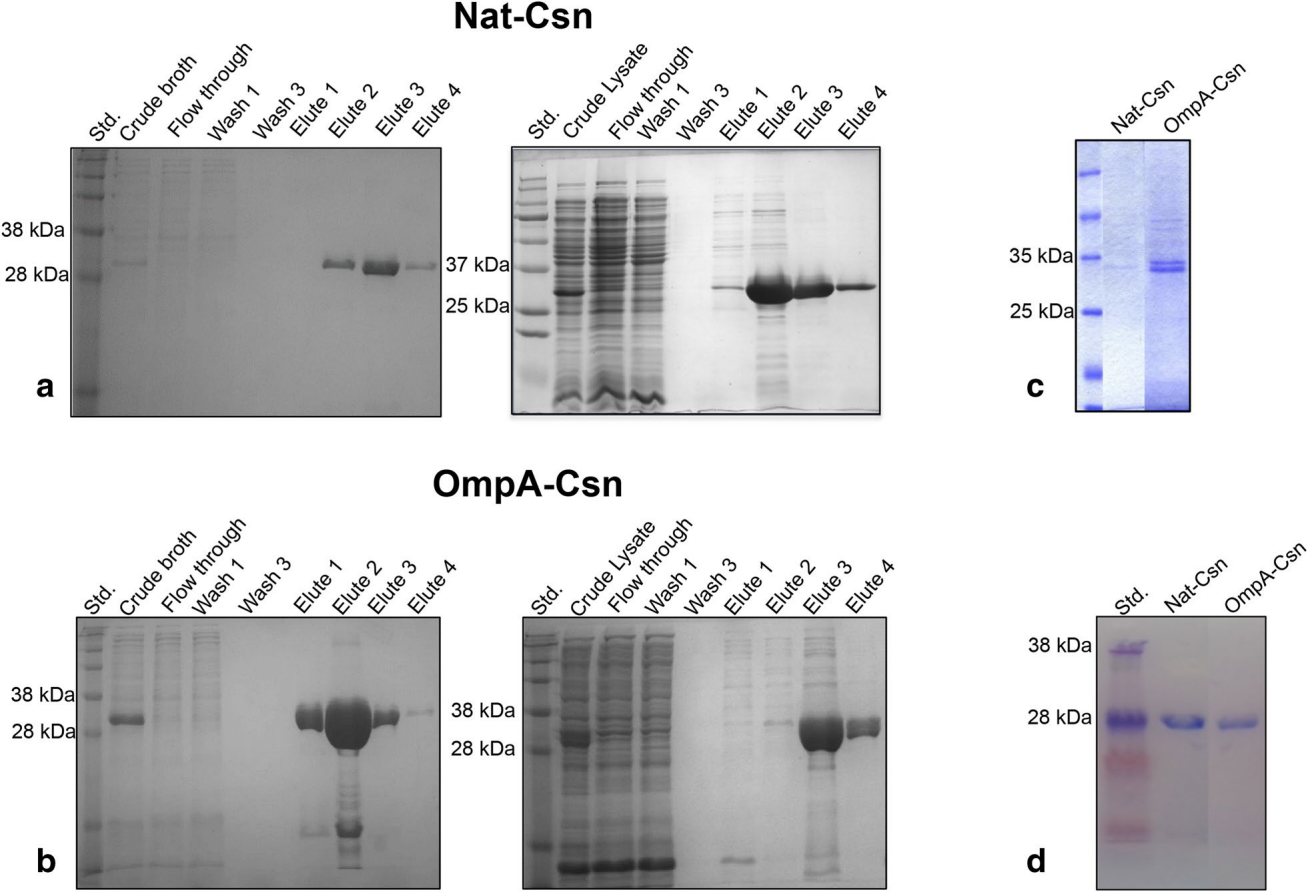
Expression and purification of secreted Nat-Csn and OmpA-Csn from culture supernatant. SDS-PAGE analysis of constructs containing native (**a**) or OmpA (**b**) signal peptides from culture supernatant (*left panel*) and cell lysate (*right panel*) at various purification steps are shown. Equal volumes (15 µL/lane) of samples were loaded into lanes, except for crude lysate, of which 5 µL/lane was loaded. **c** The comparison of crude enzymes obtained from culture broth after 20 h of induction with 0.1 mM IPTG. Equal volumes of culture broth (15 µL/lane) were loaded into *each lane*. Purified proteins from culture broth were separated by SDS-PAGE on 15 % gels and transferred onto PVDF membrane for N-terminal amino acid sequencing (**d**)

To accurately determine the yield and activity of recombinant Csn, secreted enzymes were purified from culture broth to homogeneity (Fig. 3a, b). The expected size of secreted recombinant Csn was approximately 32 kDa. The SDS-PAGE analysis of crude secreted enzymes from culture broth or cell lysate (periplasm plus cytosol) are illustrated in Fig. 3c. Routinely, approximately 18.5 and 0.4 mg/L of purified OmpA-Csn and Nat-Csn could be purified from culture supernatant of shake-flask cultivation, respectively (see Additional file 2: S7 for purification table). Both purified recombinant Csn proteins had specific activities of approximately 650 U/mg. These results are consistent with previous results on agar plates that the OmpA signal peptide is not only more efficient at directing recombinant *Bacillus* Csn secretion via the Sec-dependent pathway in an *E. coli* expression system, but it also allows for increased protein expression upon induction with IPTG.

### N-terminal sequencing of secreted recombinant Csn

**Fig. 4:**
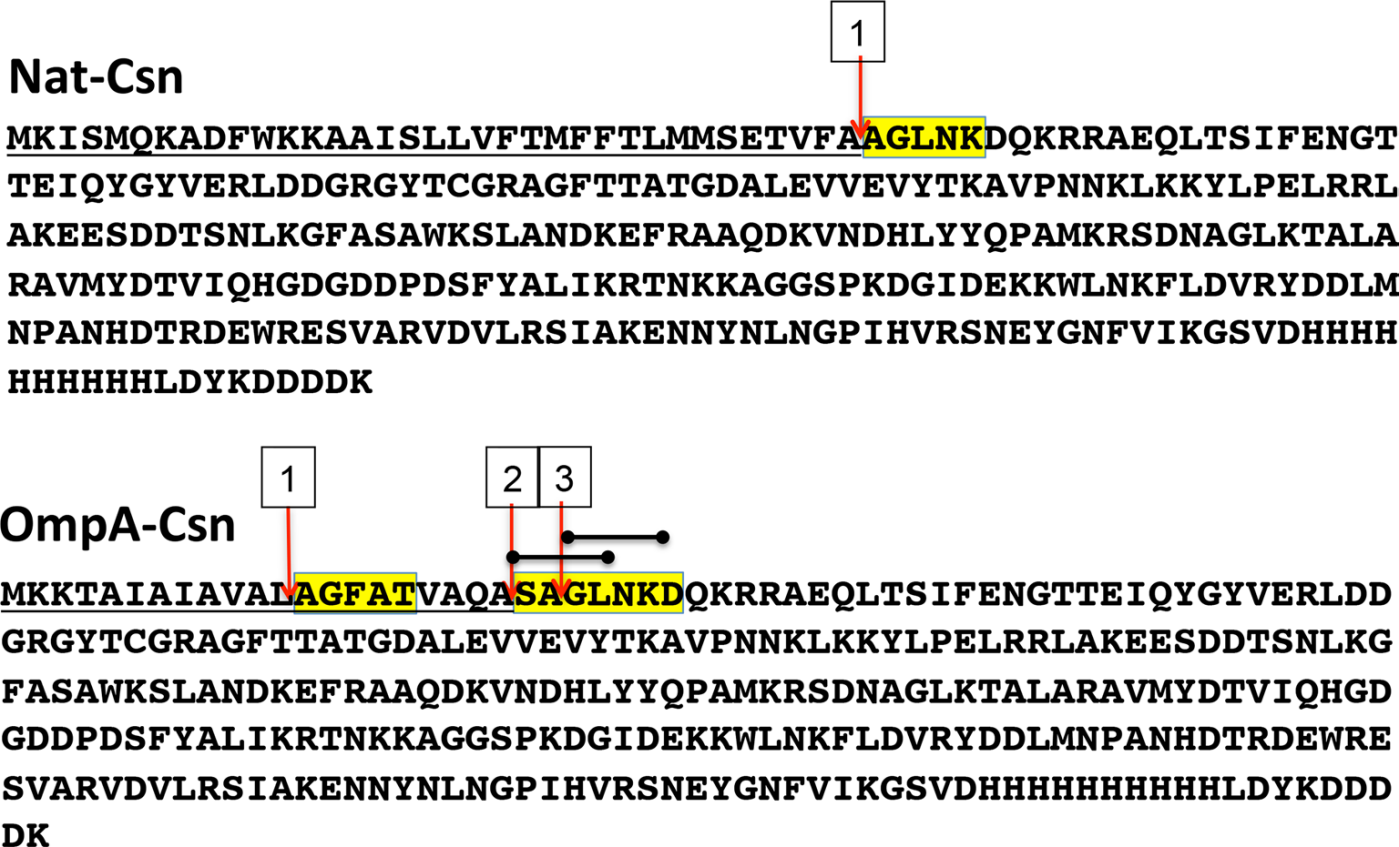
Signal peptide cleavage sites. N-terminal sequencing of Nat-Csn and OmpA-Csn was performed by Edman degradation on an Applied Biosystems Procise 492 protein sequencer. Arrows indicate cleavage sites. Native and OmpA signal peptides are underlined. The first amino acid after the underline is the predicted cleavage site. The amino acid sequences obtained from the N-terminal analysis are highlight in yellow. For Nat-Csn the sequence was AGLNK; while the three sequences from OmpA-Csn were SAGLN (*site 2*), GLNKD (*site 3*), and AGFAT (*site 1*). *Two short bars* the overlapped between *site 2* and *3*

When proteins are secreted via the *E. coli* secretory pathway, the signal peptide is cleaved by membrane-bound signal peptidases before the mature enzyme is released into the extracellular environment (Choi and Lee 2004). Since most recombinant proteins are foreign to *E. coli*, the cleavage sites can be predicted (Bendtsen et al. 2004; Zhang and Henzel 2004) but may differ from the actual cleavage sites that are often unknown. The secreted recombinant Csn were subjected to N-terminal sequencing to investigate whether changes in the structure of signal peptide could lead to alterations in amino acid sequence at the N-terminus. To do this, secreted enzymes were purified from culture broth, transferred onto PVDF (Fig. 3d) and submitted for N-terminal sequencing. The raw N-terminal sequencing data can be found in Additional file 3: S2, Additional file 4: S3, Additional file 5: S4 and Additional file 6: S5. Interestingly, our results indicated that the native signal peptide was cleaved homogenously at the expected site (see Additional file 3: S2, Additional file 4: S3), as indicated by an arrow in Fig. 4, top panel. However, when the OmpA signal peptide was used, the secreted enzyme was found to be in a heterogeneous population, whereby the signal peptide was cleaved at three different sites (see Additional file 5: S4, Additional file 6: S5). One of these sites was the predicted site for the OmpA signal peptide; however, none of these sites yielded the correct mature, native *Bacillus* enzyme (which should start at AGLN).

### MALDI-TOF mass spectrometry of secreted recombinant Csn

**Fig. 5:**
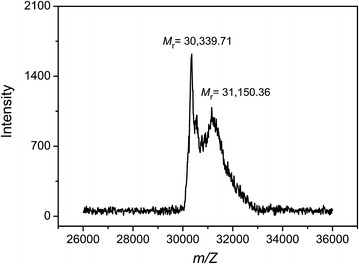
MALDI-TOF MS analysis of the recombinant OmpA-Csn. Two mass signals with molecular mass (m/z) of 30,339.71 and 31,150.36 Da as measured in a linear positive mode were indicated. Bovine insulin, equine cytochrome C, and equine apomyoglobin were used as external calibrated standard

To confirm and further analyze the heterogeneous population of Csn secreted via the OmpA signal peptide, purified secreted Csn-OmpA protein was subjected to MALDI-TOF-MS for determination of intact molecular mass. Two intense mass signals corresponding to 30,339.71 and 31,150.36 Da were detected (Fig. 5). The m/z ratio of these two signals corresponded well with amino acids 13–287 (cleavage site 1) and 22–287 (cleavage site 2), which encode proteins that are 275 and 266 amino acids, respectively. These mass determinations for purified Csn-OmpA were consistent and reproducible following several separate experiments. Since the sensitivity of MALDI TOF-MS is in the femtogram range, therefore, we could only confirm the presence of two heterogeneous products of secreted bacillus Csn using the OmpA signal peptide. Taken together these results indicated that even though the OmpA signal peptide could efficiently direct the secretion of the recombinant protein, the site of signal peptide cleavage might not have been accurate, resulting in a heterogeneous population of secreted proteins. In addition, our data indicated that the secretory machinery and the N-terminal signal peptidases of Gram-negative *E. coli* and Gram-positive *Bacillus* are highly similar, even if the structure of their cell walls are significantly different, because the *E. coli* translocation machinery could efficiently process and transport native *Bacillus* enzyme with native signal peptide.

## Discussion

Despite a substantial amount of information about secretion mechanisms, types of signal peptides and the effect of signal peptides on the secretion of different enzymes in various expression systems (Degering et al. 2010; Mathiesen et al. 2008; Nakamura et al. 1989), little information on the structure of the N-termini of the secreted recombinant proteins in *E. coli* has been reported. Even though many online databases and software programs can be used to predict signal peptides and cleavage sites, these algorithms are not always accurate (Zhang and Henzel 2004), as demonstrated in this study.

It has been previously shown that in addition to the signal peptide, the protein domain adjacent to the signal sequence, called the export initiation domain, is critical for protein translocation across the inner membrane of *E. coli* (Andersson and von Heijne 1991). *Bacillus* Csn was selected as a model in this study because it is an extracellular enzyme and therefore contains an export initiation domain that favors secretion. As such, *Bacillus* Csn was well suited for the purpose of this study, where mainly the effect of the signal peptide was compared.

While native *Bacillus* signal peptide can be recognized by *E. coli* secretion machinery, the secretion efficiency was not as high as when the OmpA signal peptide was used. In the presence of the *Bacillus* signal peptide, the secretion efficiency was 33.8 % at 20 h after induction, indicating that two-thirds of the recombinant proteins were retained in the cell. However, the N-terminal signal peptide had identical cleavage sites as in native *Bacillus* strains, indicating that N-terminal signal peptide processing is very accurate. This result indicates that both *E. coli* OmpA and native Bacillus Csn signal peptides direct the export of proteins via similar Sec-dependent secretion machinery. In addition, our data suggests that *Bacillus* and *E. coli* signal peptidases are functionally similar, even though their cell wall structures are very different. These data support the previous observation that the major components of the Sec machinery, which are required for protein secretion in both *Bacillus* and *E. coli*, are quite similar (Yuan et al. 2010).

Both gram-positive and gram-negative employ a similar type I signal peptidase (SPase), a membrane-bound endopeptidase, to remove the signal peptide from exported pre-enzymes during the late stages of the transport process (Paetzel 2014; van Roosmalen et al. 2004). The consensus SPase recognition sequence is Ala-X-Ala at positions-1 and -3 relative to the cleavage site in pre-proteins (Paetzel 2014). *B. subtilis* proteomics analysis revealed that 71 % of the corresponding signal peptides contain the consensus Ala-X-Ala recognition sequence; while 18 % of the identified extracellular proteins contain a Val-X-Ala recognition sequence (van Roosmalen et al. 2004). Therefore, native Bacillus SP could be cleaved precisely after VFA sequence, generating precise N-terminus as indicated in Fig. 4. As for construct containing OmpA signal peptide, there were two Ala-X-Ala sequences, which was artificially created by genetic engineering, consequently we detected two cleavage sites after the two Ala-X-Ala sequences (cleavage site 2 and 3). The cleavage site 1 was unusual as it was situated in the middle of hydrophobic region of signal peptide, before a helix-breaking glycine residue. This cleavage might have occurred via a different secretory pathway due to the incompatibility between the export initiation domain of *Bacillus* Csn and Gram-negative OmpA signal peptide. This result suggested that, one should avoid creating more than one Ala-X-Ala sequence at the junction between signal peptide and mature protein, when engineering a recombinant protein for secretory production.

The two signal peptides had significantly different effects on the secretory production of recombinant proteins in *E. coli*. As shown in Table 1, the OmpA signal peptide led to significantly higher yields of recombinant enzyme, compared with the native signal peptide. Codon optimization analysis, using OPTIMIZER, an online application for improving expression levels, indicated that the codon adaptation index (CAI) based on codon usage of predicted highly expressed genes of the N-terminus of the native *Bacillus* signal peptide, was only 0.537 (1.0 is the highest) (Puigbo et al. 2007). Therefore one explanation for the higher expression yield could be because the codon of the homologous OmpA sequence located at the 5′ end of the gene is more compatible with the *E. coli* translation machinery, especially at the initiation of translation step. Further study by codon optimization of native Csn signal peptide to mimic the codon usage of *E. coli* will be necessary to test if this could help improve expression levels without compromising the fidelity of signal peptide cleavage. If successful, this may have broader commercial applications in the future.

It is interesting to note that the p*tac* promoter was leaky (de Boer et al. 1983), resulting in low protein expression in the absence of induction with IPTG. At this low expression level, both signal peptides had 80 % secretion efficiency. It is possible that the secretion machinery was not fully occupied and still able to process the secretion of both signal peptides (Driessen et al. 2001). However, when IPTG was used to induce protein over-expression, secretion mediated by the native *Bacillus* signal peptide, which was less compatible with the *E. coli* translocase complex, became the rate-limiting step for secretory production much earlier than when OmpA was used (de Keyzer et al. 2003). Another possibility is that the signal peptides used different secretion pathways (Muller et al. 2001). Recently, it was shown that a short peptide could serve as a signal peptide and guide heterologous cellulose proteins across both the inner and outer membranes of *E. coli* (Gao et al. 2015). Taken together these results suggest that when the N-terminal sequence of a protein is not critical, the OmpA signal peptide is preferred for the secretion of recombinant proteins in *E. coli*-based systems.

## Conclusions

Our results indicated that in an *E. coli* expression system, the *E. coli* OmpA signal peptide was more efficient than the native *Bacillus* signal peptide, for both expression and secretion of *Bacillus* Csn; however, cleavage of the signal peptide was not precise. Moreover, our results also indicated that the secretion machinery of Gram-negative *E. coli* could be used to correctly process the signal sequence and efficiently direct the secretion of extracellular hydrolytic enzymes from Gram-positive bacteria, despite significant differences between the cell walls of Gram-positive and Gram-negative bacteria. These results can be used for the engineering of other recombinant proteins for secretory production in *E. coli*.

## Experimental procedures

### Bacterial strains and plasmids

*Bacillus subtilis* strain 168 (ATCC23857) was obtained from the American Type Culture Collection. The bacteria were grown on NA agar at 30 °C. *E. coli* DH5α (Life Technologies, USA) and TOP10 (Invitrogen, USA) were used as a cloning and expression host, respectively. The plasmid pMY202, which was used for cloning and expression of the *B. subtilis* chitosanase gene, was modified from pFLAG-CTS (Sigma, USA) by replacing the multiple cloning sites (MCS) between *Hind*III and *Sal*I of the pFLAG-CTS in a way that the *Sal*I was destroyed after ligation (Yamabhai et al. 2011). The map of pMY202 can be found in Additional file 7: S1.

### Substrate

Low molecular weight Csn [product number 448869 (75–85 % DDA)] was purchased from Sigma-Aldrich and soluble chitosan (10 mg/mL) was prepared by dissolving 10 g of chitosan in 400 mL of distilled water and 90 mL of 1 M acetic acid. The Csn solution was adjusted to pH 5.5 with 1 M sodium acetate to a final volume of 1 L with distilled water.

### Molecular cloning

The genes encoding Csn from *B. subtilis* 168 containing the native signal peptide (Nat-Csn) or the mature enzyme fused with the *E. coli* OmpA signal peptide (OmpA-Csn) were cloned by PCR-based methods into the pMY202 vector (Additional file 7: S1), according to a previously published protocol (Songsiriritthigul et al. 2010). The primers were designed from the published database of the DNA sequence of the Csn gene of *B. subtilis* 168 (NCBI accession number: NC_000964 REGION: complement (2747984..2748817). The primers, B.subCsnNdeIFw: 5′ CTG TGC CAT ATG AAA ATC AGT ATG CAA AAA GCA GAT TTT TGG 3′ and B.subCsnBamHIRv: 5′ GCA CAG GGA TCC TTT GAT TAC AAA ATT ACC GTA CTC GTT TGA AC 3′ were used for PCR amplification of the *B. subtilis* Csn gene containing the native signal peptide (Nat-Csn). The PCR products were cut with *Nd*eI and *Bgl*II and cloned into *Nd*eI and *Bgl*II sites on the pMY202 plasmid. For the construction of the recombinant chitosanase gene, of which the native signal peptide was replaced with the *E. coli* OmpA signal peptide (OmpA-Csn), primers B.subCsnOmpAHindIIIFw: 5′ CTGTGCAAG CTT CGG CGG GAC TGA ATA AAG ATC AAA AGC3′ and B.subCsnBamHIRv: 5′ GCA CAG GGA TCC TTT GAT TAC AAA ATT ACC GTA CTC GTT TGA AC 3′ were used in the PCR reaction. The PCR products were cut with *Hind*III and *BamH*I and ligated into a pMY202 vector that had been digested with the same enzymes. The recombinant constructs of *B. subtilis* Csn containing either the native or the OmpA signal peptide were designated Nat-Csn/pMY202 and OmpA-Csn/pMY202, respectively. The integrity of the constructs was confirmed by automated DNA sequencing (Macrogen, Korea).

### Expression and preparation of recombinant chitosanases from various compartments

Four colonies of freshly transformed *E. coli* TOP10 harboring appropriate constructs were transferred into 20 mL Luria–Bertani (LB) broth containing 100 µg/mL ampicillin (LB-Amp) and grown overnight at 37 °C. Then, 2 % of the overnight cultures were added into 200 mL LB-Amp broth and grown at 37 °C with shaking until an optical density at 600 nm (OD_600_) of 0.6–0.7 was reached. Isopropyl-β-d-thiogalactopyranoside (IPTG) was added into the culture broth to a final concentration of 0.1 mM (for purification as shown in Fig. 3) or 0.5 mM (for determination of enzyme activities in various compartments as reported in Table 1), and the incubation was continued at ambient temperature (27–28 °C) with shaking. Fifty mL of culture was collected after induction for 4 and 20 h and then centrifuged at 4000×*g* for 30 min at 4 °C. Preparation of periplasmic extract and cell lysate (cytosolic fraction) was done as previously described (Yamabhai et al. 2008).

### SDS-PAGE

Denaturing sodium dodecylsulfate-polyacrylamide gel electrophoresis (SDS-PAGE) was performed according to the method of Laemmli (Laemmli 1970). Protein bands were stained by Coomassie brilliant blue R-250. Protein ladder (10–200 kDa) was purchased from Fermentas (St. Leon, Germany) and Bio-Rad. The protein samples were briefly heated (3 min) in loading buffer (Laemmli buffer) with reducing agent (100 mM β-mercaptoethanol) at 100 °C, using a heat block before loading onto the gel.

### Enzyme activity assay on agar plates

The activity of recombinant Csn was assayed on LB agar plates containing 100 µg/mL ampicillin and 0.1 % (w/v) of low MW chitosan. Various concentrations of IPTG (0, 0.1, 0.5 mM) were spread onto the plate before freshly transformed cells were spotted onto the plates and incubated at 37 °C. Hydrolytic clear zones were observed and the diameters of the clear zones were measured at various time points. The experiments were done in triplicate. The average diameters with SD values were reported.

### Chitosanase activity assay

Standard Csn activity was determined using the 3,5-dinitrosalicylic acid (DNS) method (Miller 1959). The reaction mixture consisted of 40 µL of enzyme (0.4 µg) and 160 µL of 0.5 % (w/v) of soluble chitosan in 200 mM sodium acetate buffer, pH5.5. The reaction was incubated in a Thermomixer Comfort (Eppendorf AG, Hamburg, Germany) at 50 °C for 5 min, with mixing at 900 rpm. The reaction was stopped by adding 200 µL of DNS solution and centrifuged at 12,000×*g* for 5 min to precipitate the remaining chitosan. Then, the color was developed by heating at 100 °C for 20 min and cooled on ice. The concentration of reducing sugar in the supernatant was determined by measuring the OD at 540 nm, using d-glucosamine (1–5 µmol/mL) as a standard. One unit of Csn activity was defined as the amount of enzyme that released 1 µmol of D-glucosamine per minute under standard assay conditions. The experiments were performed in duplicate.

### Purification of recombinant chitosanase

Recombinant 10× His-tagged Csn proteins were purified from culture supernatant and cell lysate by immobilized metal affinity chromatography (IMAC), using Ni–NTA agarose (Qiagen, Germany) as previously described (Juajun et al. 2011). The enzyme was eluted with 250 mM imidazole. The eluted enzyme was passed through Vivaspin6 columns (M_r_cut-off 10 kDa; GE Healthcare, Sweden) to remove imidazole and concentrate the protein. The purified enzyme was stored at 4 °C until further use. Protein concentrations were determined by the method of Bradford (Bradford 1976) using a Bio-Rad protein assay kit and bovine serum albumin (BSA) as the standard. The standard calibration curve was constructed from 0.05 to 0.5 mg/mL BSA.

### N-terminal sequencing

1.25 µg samples of purified OmpA-Csn and Nat-Csn were separated by SDS-PAGE on 15 % gels and electroblotted onto polyvinylidenedifluoride (PVDF) membrane (Bio-Rad, USA) in 50 mM borate buffer containing 10 % (v/v) methanol, pH 9. After blotting, the membrane was stained with Coomassie blue for 3 min, followed by destaining of the membrane with destaining solution (40 % (v/v) methanol and 10 % (v/v) acetic acid). N-terminal sequences were commercially analyzed using Edman degradation on an Applied Biosystems Procise 492 protein sequencer (Protein Micro-Analysis Facility, Medical University of Innsbruck, Austria).

### MALDI-TOF MS

Protein samples were loaded into Zeba™ Spin Desalting Columns (Thermo Scientific Inc., USA) pre-equilibrated with water. The columns were centrifuged at 1500 rpm for 2 min and the desalted fraction was precipitated overnight with 2 volumes of cold acetone at −20 °C After centrifugation at 12,000 rpm for 15 min, the protein pellet was resuspended in 0.1 %TFA/50 %ACN to a final concentration of 10 μg/μL. The protein was mixed with MALDI matrix solution (10 mg sinapinic acid in 1 mL of 50 % acetonitrile containing 0.1 % trifluoroacetic acid), directly spotted onto the MALDI target (MTP 384 ground steel, Bruker Daltonik, GmbH), and allowed to dry at room temperature. MALDI-TOF MS spectra were collected using Ultraflex III TOF/TOF (Bruker Daltonik, GmbH) in linear positive mode with a mass range of 5000–100,000 Da. Five hundred shots were accumulated with a 200-Hz laser for each sample. MS spectra were analyzed by FlexAnalysis software (Bruker Daltonik, GmbH). Bovine insulin, equine cytochrome C and equine apomyoglobin were used as external protein calibrations.

## Additional files

10.1186/s40064-016-2893-y Clear Zones from an agar plate assay.
10.1186/s40064-016-2893-y Purification table.
10.1186/s40064-016-2893-y Summary of N-terminal sequence analysis of secreted Nat-Csn.
10.1186/s40064-016-2893-y Summary of N-terminal sequence analysis of secreted OmpA-Csn.
10.1186/s40064-016-2893-y Raw data of N-terminal sequence analysis of secreted Nat-Csn.
10.1186/s40064-016-2893-y Raw data of N-terminal sequence analysis of secreted Nat-Csn.
10.1186/s40064-016-2893-y Map of pMY202 expression vector. The pMY202 vector was used for the expression of the *Bacillus* chitosanase gene. This plasmid contained the *E. coli* OmpA signal peptide (blue) upstream from the multiple cloning site (MCS). Protein expression was under the control of a *tac* promoter. The vector contained an ampicillin resistance gene and a C-terminal 10×-histidine tag (red) to facilitate affinity purification. The plasmid used an origin of replication (ori) from pBR322, which has a low-copy number (15–20 plasmids per cell).

## References

[CR1] Albiniak AM, Matos CF, Branston SD, Freedman RB, Keshavarz-Moore E, Robinson C (2013). High-level secretion of a recombinant protein to the culture medium with a *Bacillus subtilis* twin-arginine translocation system in *Escherichia coli*. FEBS J.

[CR2] Andersson H, von Heijne G (1991). A 30-residue-long “export initiation domain” adjacent to the signal sequence is critical for protein translocation across the inner membrane of *Escherichia coli*. Proc Natl Acad Sci U S A.

[CR3] Bendtsen JD, Nielsen H, von Heijne G, Brunak S (2004). Improved prediction of signal peptides: SignalP 3.0. J Mol Biol.

[CR4] Bradford MM (1976). A rapid and sensitive method for the quantitation of microgram quantities of protein utilizing the principle of protein-dye binding. Anal Biochem.

[CR5] Choi JH, Lee SY (2004). Secretory and extracellular production of recombinant proteins using *Escherichia coli*. Appl Microbiol Biotechnol.

[CR6] de Boer HA, Comstock LJ, Vasser M (1983). The tac promoter: a functional hybrid derived from the trp and lac promoters. Proc Natl Acad Sci USA.

[CR7] de Keyzer J, van der Does C, Driessen AJ (2003). The bacterial translocase: a dynamic protein channel complex. Cell Mol Life Sci.

[CR8] Degering C, Eggert T, Puls M, Bongaerts J, Evers S, Maurer KH, Jaeger KE (2010). Optimization of protease secretion in *Bacillus subtilis* and *Bacillus licheniformis* by screening of homologous and heterologous signal peptides. Appl Environ Microbiol.

[CR9] Driessen AJ, Manting EH, van der Does C (2001). The structural basis of protein targeting and translocation in bacteria. Nat Struct Biol.

[CR10] Gao D, Wang S, Li H, Yu H, Qi Q (2015). Identification of a heterologous cellulase and its N-terminus that can guide recombinant proteins out of *Escherichia coli*. Microb Cell Fact.

[CR11] Juajun O, Nguyen TH, Maischberger T, Iqbal S, Haltrich D, Yamabhai M (2011). Cloning, purification, and characterization of beta-galactosidase from *Bacillus licheniformis* DSM 13. Appl Microbiol Biotechnol.

[CR12] Laemmli UK (1970). Cleavage of structural proteins during the assembly of the head of bacteriophage T4. Nature.

[CR13] Mathiesen G, Sveen A, Piard JC, Axelsson L, Eijsink VG (2008). Heterologous protein secretion by *Lactobacillus plantarum* using homologous signal peptides. J Appl Microbiol.

[CR14] Mergulhao FJ, Summers DK, Monteiro GA (2005). Recombinant protein secretion in *Escherichia coli*. Biotechnol Adv.

[CR15] Miller GL (1959). Use of dinitrosalicylic acid reagent for determination of reducing sugar. Anal Chem.

[CR16] Muller M, Koch HG, Beck K, Schafer U (2001). Protein traffic in bacteria: multiple routes from the ribosome to and across the membrane. Prog Nucleic Acid Res Mol Biol.

[CR17] Nakamura K, Fujita Y, Itoh Y, Yamane K (1989). Modification of length, hydrophobic properties and electric charge of *Bacillus subtilis* alpha-amylase signal peptide and their different effects on the production of secretory proteins in *B. subtilis* and *Escherichia coli* cells. Mol Gen Genet.

[CR18] Paetzel M (2014). Structure and mechanism of *Escherichia coli* type I signal peptidase. Biochim Biophys Acta.

[CR19] Pechsrichuang P, Yoohat K, Yamabhai M (2013). Production of recombinant *Bacillus subtilis* chitosanase, suitable for biosynthesis of chitosan-oligosaccharides. Bioresour Technol.

[CR20] Puigbo P, Guzman E, Romeu A, Garcia-Vallve S (2007). OPTIMIZER: a web server for optimizing the codon usage of DNA sequences. Nucleic Acids Res.

[CR21] Rinas U, Hoffmann F (2004). Selective leakage of host-cell proteins during high-cell-density cultivation of recombinant and non-recombinant *Escherichia coli*. Biotechnol Prog.

[CR22] Songsiriritthigul C, Lapboonrueng S, Pechsrichuang P, Pesatcha P, Yamabhai M (2010). Expression and characterization of *Bacillus licheniformis* chitinase (ChiA), suitable for bioconversion of chitin waste. Bioresour Technol.

[CR23] van Roosmalen ML, Geukens N, Jongbloed JD, Tjalsma H, Dubois JY, Bron S, van Dijl JM, Anne J (2004). Type I signal peptidases of Gram-positive bacteria. Biochim Biophys Acta.

[CR24] Yamabhai M, Emrat S, Sukasem S, Pesatcha P, Jaruseranee N, Buranabanyat B (2008). Secretion of recombinant *Bacillus* hydrolytic enzymes using *Escherichia coli* expression systems. J Biotechnol.

[CR25] Yamabhai M, Buranabanyat B, Jaruseranee N, Songsiriritthigul C (2011). Efficient *E. coli* expression systems for the production of recombinant beta-mannanases and other bacterial extracellular enzymes. Bioeng Bugs.

[CR26] Yuan J, Zweers JC, van Dijl JM, Dalbey RE (2010). Protein transport across and into cell membranes in bacteria and archaea. Cell Mol Life Sci.

[CR27] Zhang Z, Henzel WJ (2004). Signal peptide prediction based on analysis of experimentally verified cleavage sites. Protein Sci.

[CR28] Zhou Z, Zhao S, Wang S, Li X, Su L, Ma Y, Li J, Song J (2015). Extracellular overexpression of chitosanase from *Bacillus* sp. TS in *Escherichia coli*. Appl Biochem Biotechnol.

