# Supplementary material for: OmpA signal peptide leads to heterogenous secretion of B. subtilis chitosanase enzyme from E. coli expression system
Source: Springerplus. 2016 Jul 28;5(1):1200. doi: 10.1186/s40064-016-2893-y (PMC4963352; doi:10.1186/s40064-016-2893-y)
Supplement: Supplementary file 1 — 10.1186/s40064-016-2893-y Clear Zones from an agar plate assay. [file 40064_2016_2893_MOESM1_ESM.pdf]

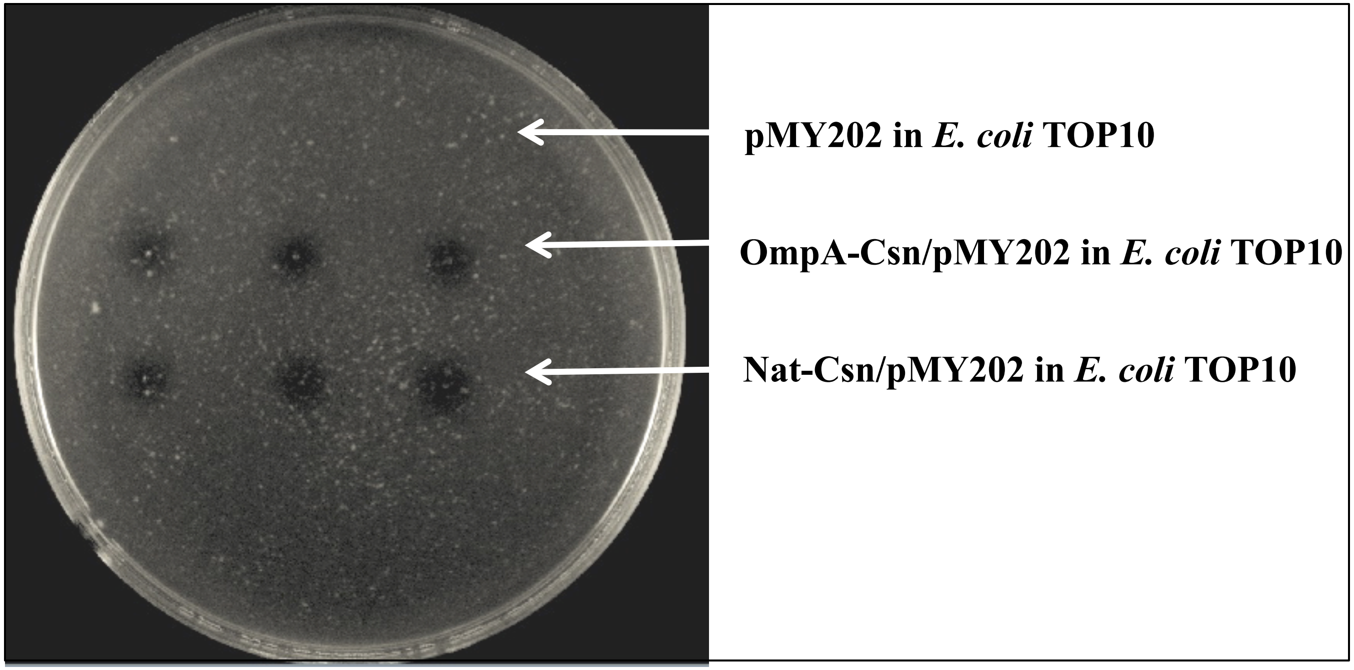
pMY202 in E. coli TOP10
OmpA-Csn/pMY202 in E. coli TOP10
Nat-Csn/pMY202 in E. coli TOP10

Figure2

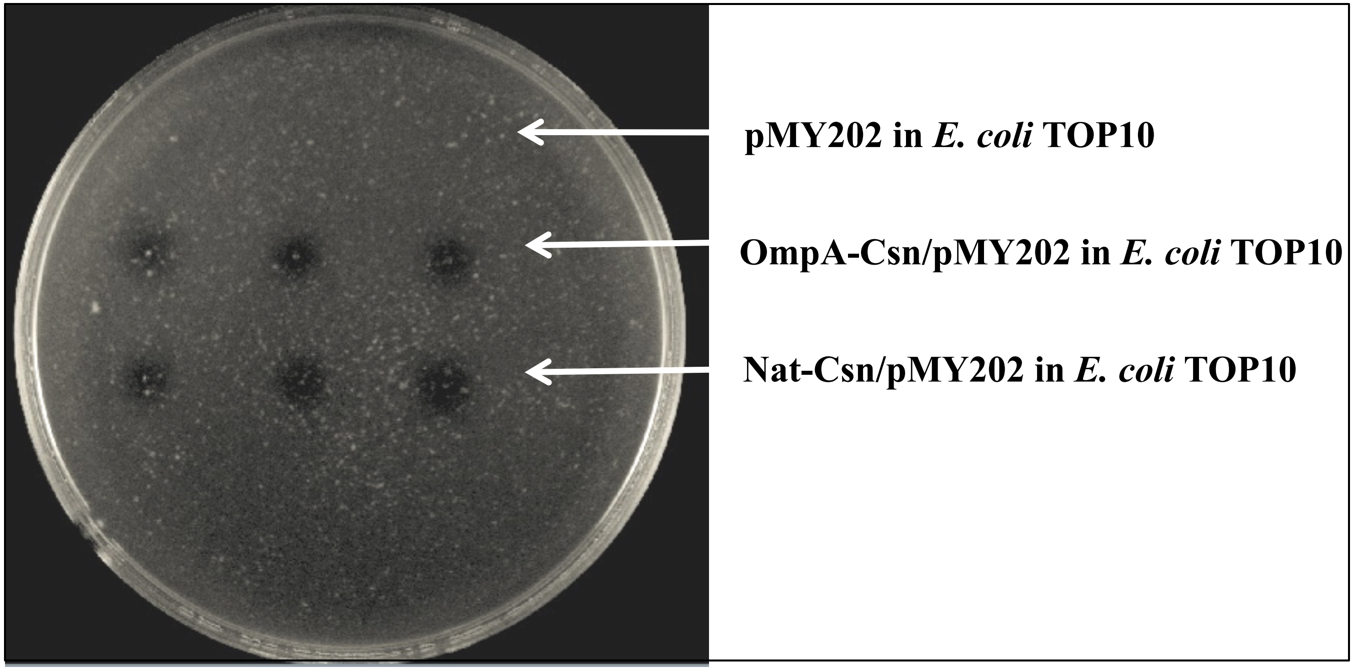

Please note that the texture of a chitosan plate for testing chitosanase activity was not smooth and homogeneous. There were a number of white spots of non-soluble chitosan. Even if this did not interfere with clear zone formation, it was very difficult to spot the bacterial colony, which was very small (E. coli Top10 colony is very small). Therefore, the diameters of the clear zone were measured based on the longest line between the rims of the circular clear zone (diameter). The measurement was done in triplicate as illustrated.

Supplementarty Material S6
