# Supplementary material for: OmpA signal peptide leads to heterogenous secretion of B. subtilis chitosanase enzyme from E. coli expression system
Source: Springerplus. 2016 Jul 28;5(1):1200. doi: 10.1186/s40064-016-2893-y (PMC4963352; doi:10.1186/s40064-016-2893-y)
Supplement: Supplementary file 2 — 10.1186/s40064-016-2893-y Purification table. [file 40064_2016_2893_MOESM2_ESM.pdf]

Supplementary Material S7

Purification table

Sample	Protein mg/mL	Total protein mg/L	Activity (U/mL)	Total activity U/L	Specific act. (U/mg)	Purity (fold)	%Yield
Csn-OmpA							
Crude broth	0.22	216	48.7	48700	225	1.00	100
Purify broth	2.64	18.5	1370	9600	519	2.31	19.7
Crude Lysate	1.20	60.2	140	7020	117	1.00	100
Purify Lysate	0.56	2.9	253	1301	456	3.92	18.5
Csn-Native							
Crude broth	0.09	89.4	6.1	6095	68	1.00	100
Purify broth	0.14	0.38	37.9	106	280	4.10	1.74
Crude Lysate	3.68	184	704	35200	191	1.00	100
Purify Lysate	1.49	5.97	530	2120	355	1.86	6.02

The culture volume in this experiment was 300 ml. The enzyme was induced for over expression with 0.1 mM IPTG.
