# Supplementary material for: OmpA signal peptide leads to heterogenous secretion of B. subtilis chitosanase enzyme from E. coli expression system
Source: Springerplus. 2016 Jul 28;5(1):1200. doi: 10.1186/s40064-016-2893-y (PMC4963352; doi:10.1186/s40064-016-2893-y)
Supplement: Supplementary file 3 — 10.1186/s40064-016-2893-y Summary of N-terminal sequence analysis of secreted Nat-Csn. [file 40064_2016_2893_MOESM3_ESM.pdf]

A. Univ.-Prof. Dr. Herbert Lindner
Sektion für Klinische Biochemie-Biozentrum
PROTEINSEQUENZIERUNG
Innrain 80-82
A-6020 Innsbruck

Tel.: (0512)-9003-70310
Fax : (0512)-9003-73300
herbert.lindner@i-med.ac.at

Analysenergebnis

Sequencer: ABI-Procise 492		*LAUF-Nr.*	13015
PROBE :	Csn_native B 22.5.13	*Datum*	22.5.13
ID	Dietmar_Haltrich_150513	*Tel.*	

Peptid	Protein	PVDF	GF	BIOPR	BLOT	Menge	MW	I.Y. pM
☐	■	■	☐	☐	■	pM	34kDa	R.Y %

**AS**	Haupt- Sequenz	Neben- Sequenz	**AS**	Haupt- Sequenz	Neben Sequenz	**AS**	Haupt- Sequenz	Neben-. Sequenz
1	**A**	*DSG*	16			31		
2	**G**		17			32		
3	**L**		18			33		
4	**N**		19			34		
5	**K**		20			35		
6			21			36		
7			22			37		
8			23			38		
9			24			39		
10			25			40		
11			26			41		
12			27			42		
13			28			43		
14			29			44		
15			30			45		

Kommentar Sequence unambiguous. Sample is pure.
Sequence starts at position 23 of the protein.

OP BS

Dr. H. LINDNER
