# Supplementary figures and images for: OmpA signal peptide leads to heterogenous secretion of B. subtilis chitosanase enzyme from E. coli expression system
Source: Springerplus. 2016 Jul 28;5(1):1200. doi: 10.1186/s40064-016-2893-y (PMC4963352; doi:10.1186/s40064-016-2893-y)

Std. 2pM

Cycle 2: 3.0

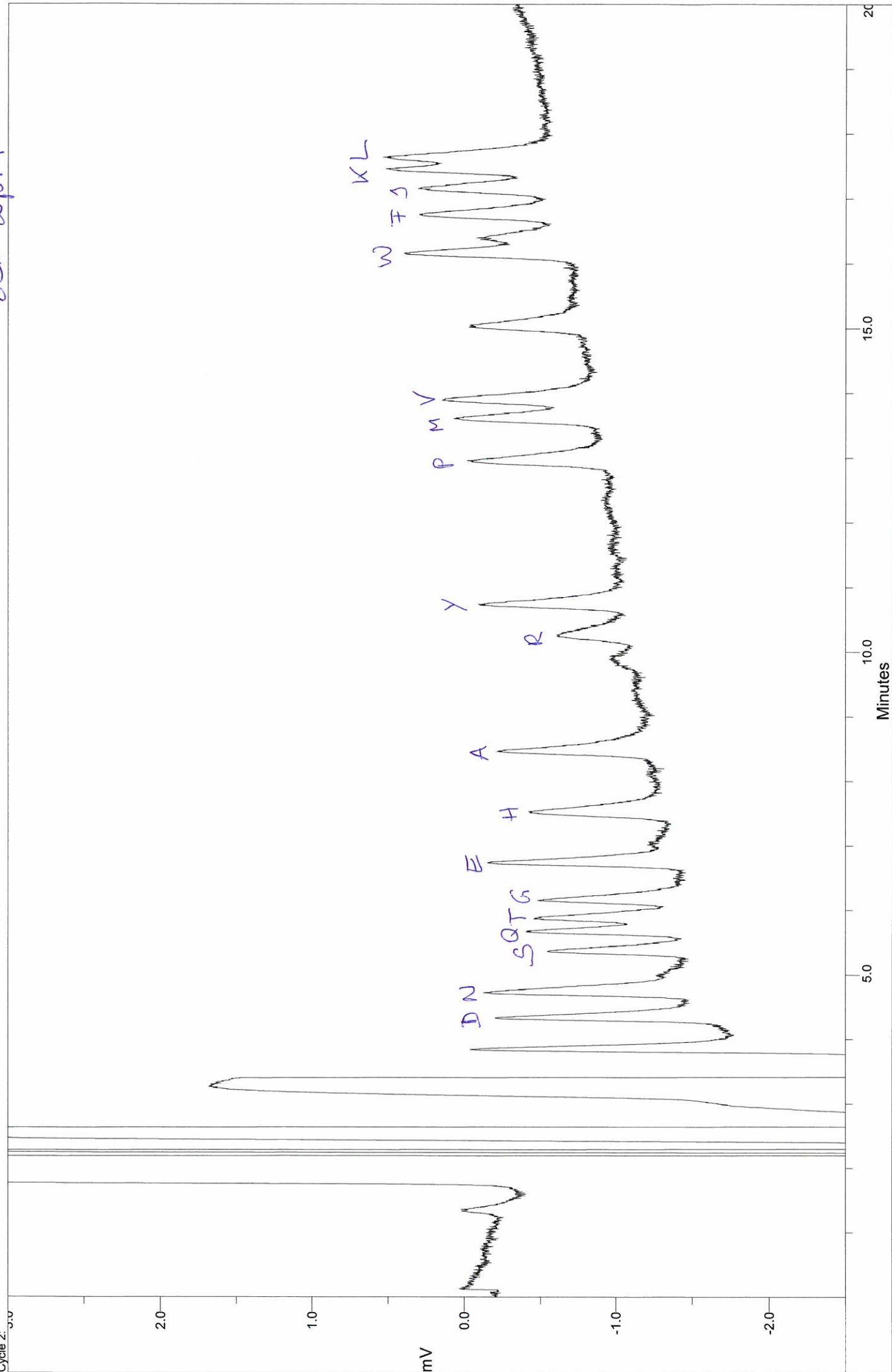

Cycle 3:

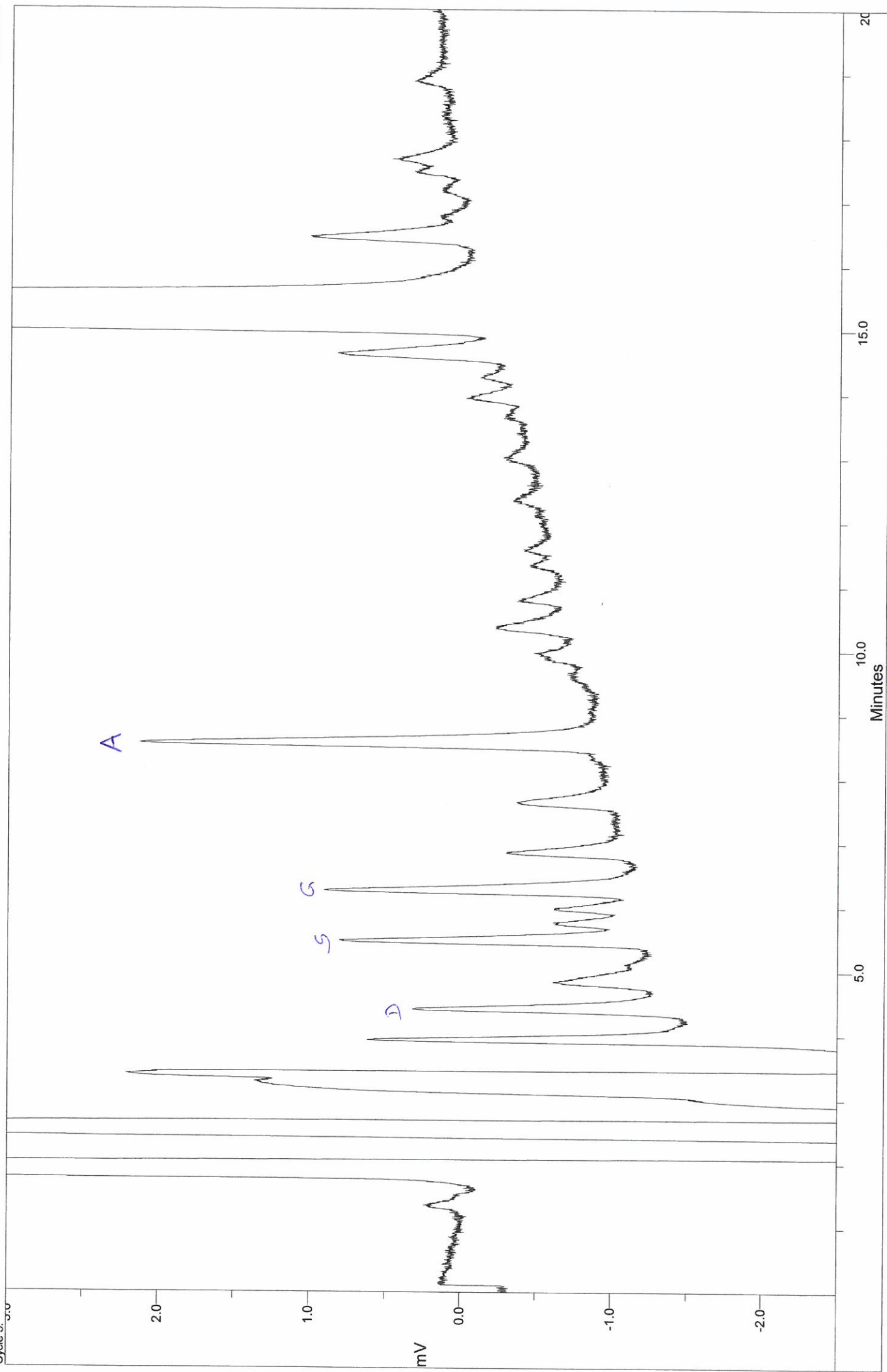

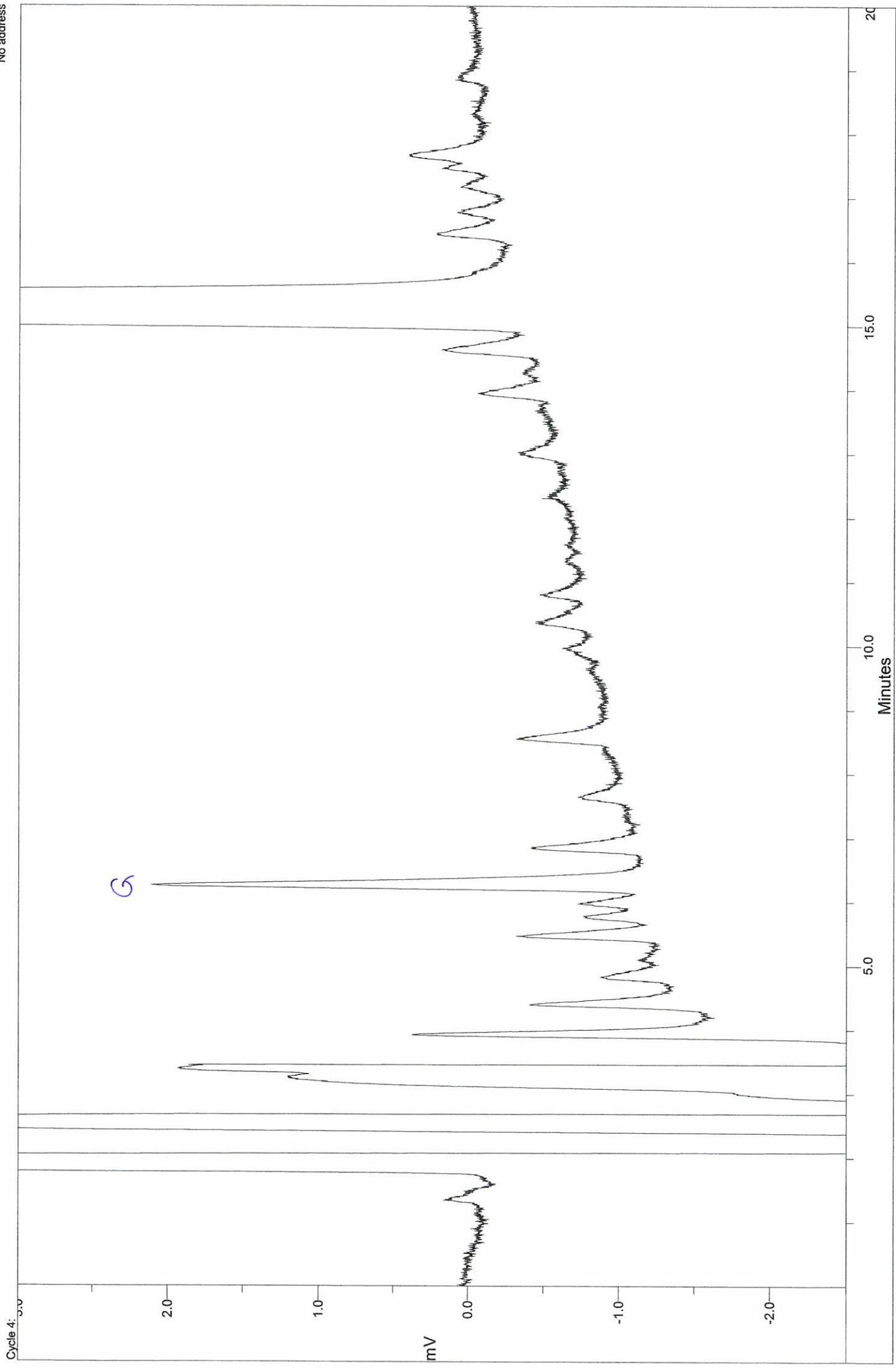

Cycle 5:

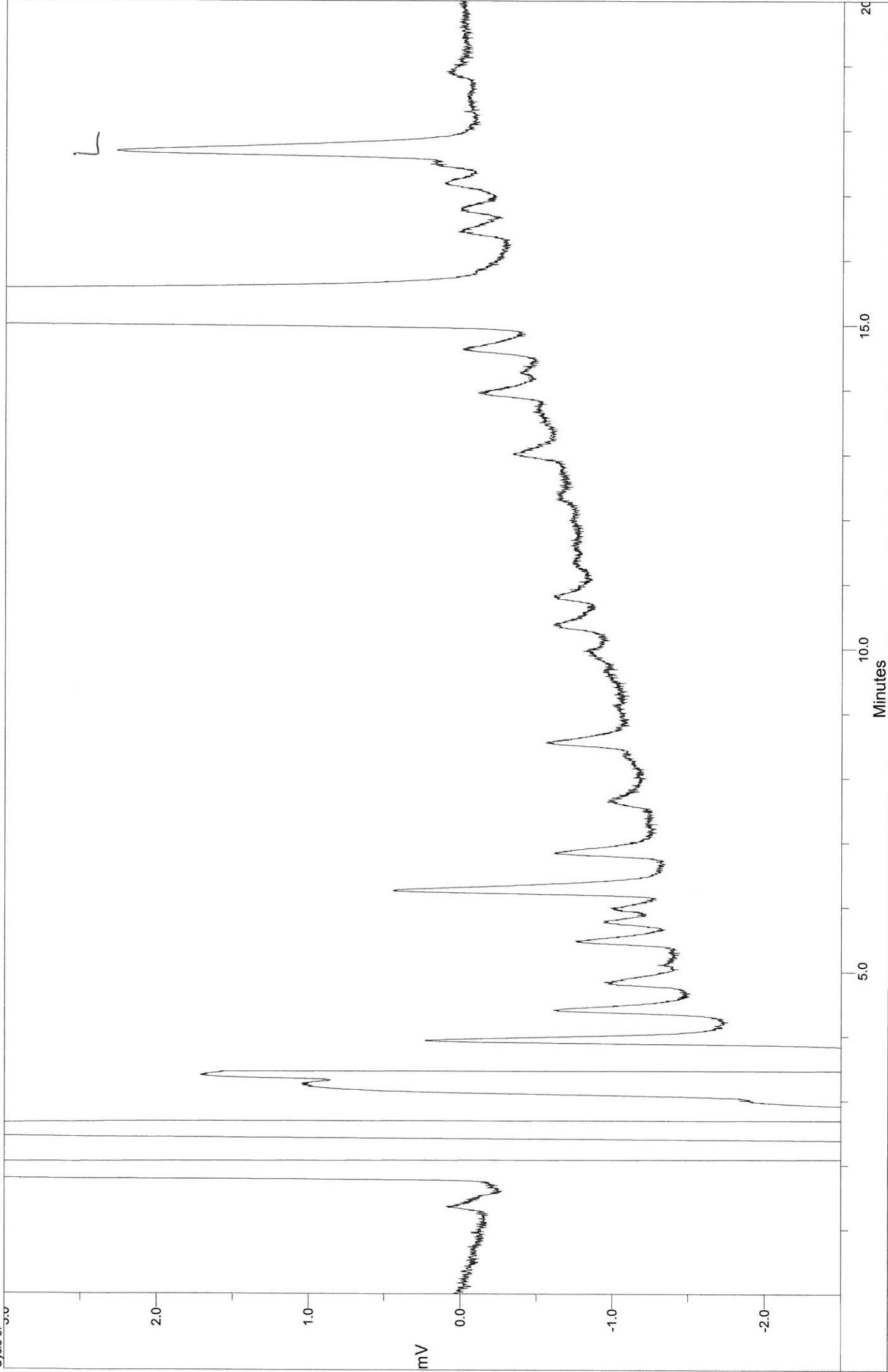

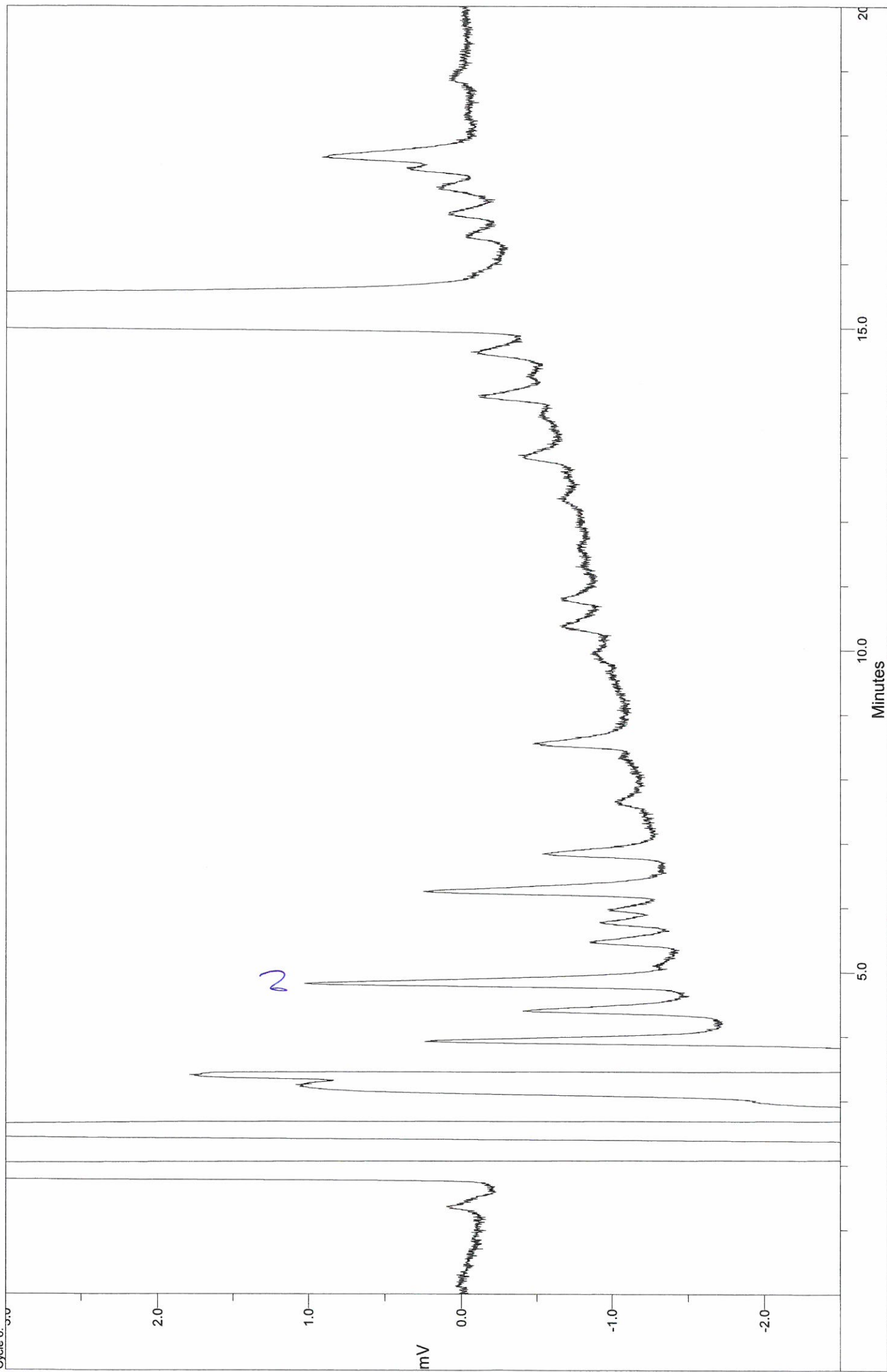

Cycle 7:

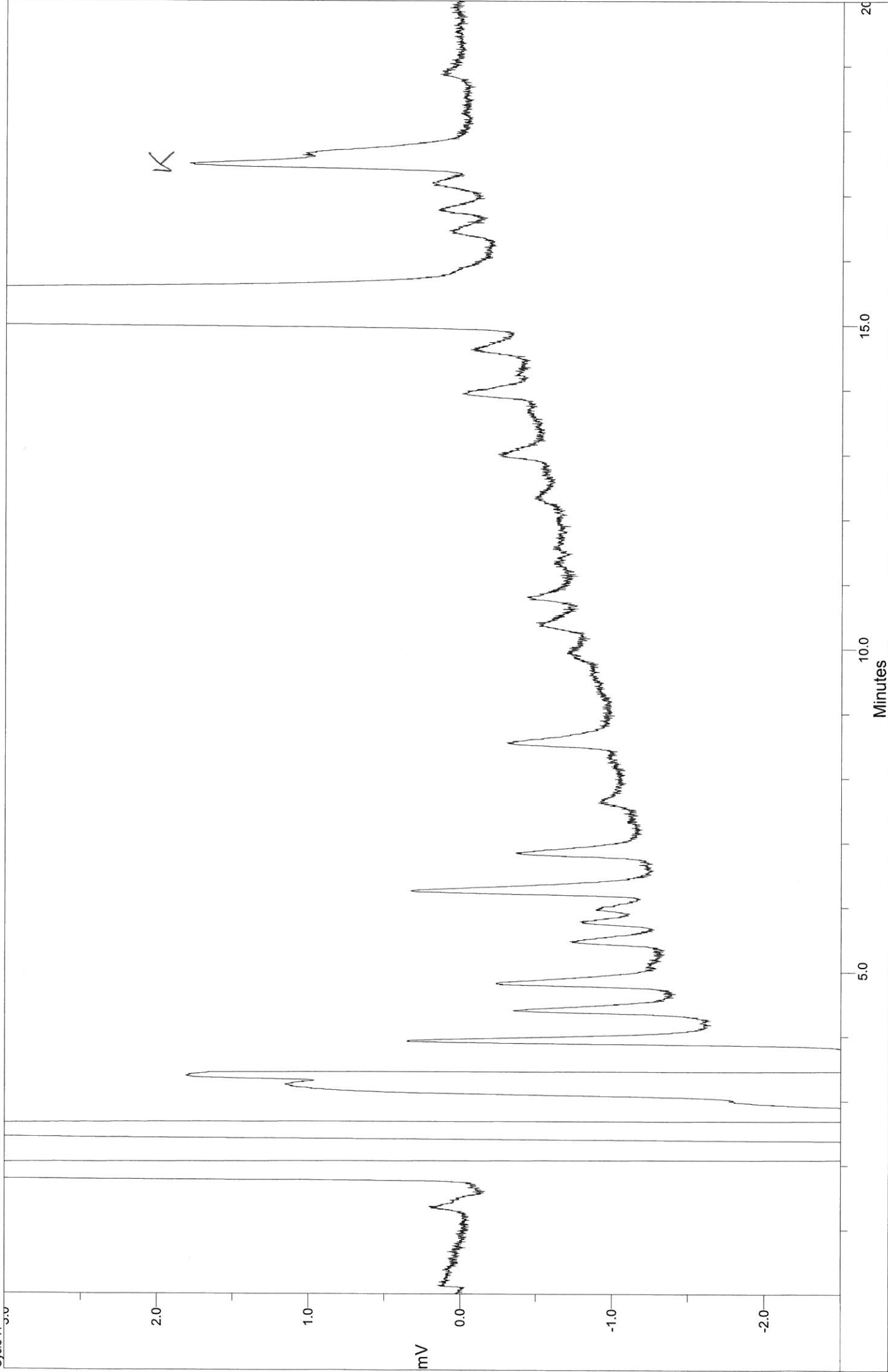

Supplement: Supplementary file 4 — 10.1186/s40064-016-2893-y Summary of N-terminal sequence analysis of secreted OmpA-Csn. [file 40064_2016_2893_MOESM4_ESM.pdf]

80. 2pM

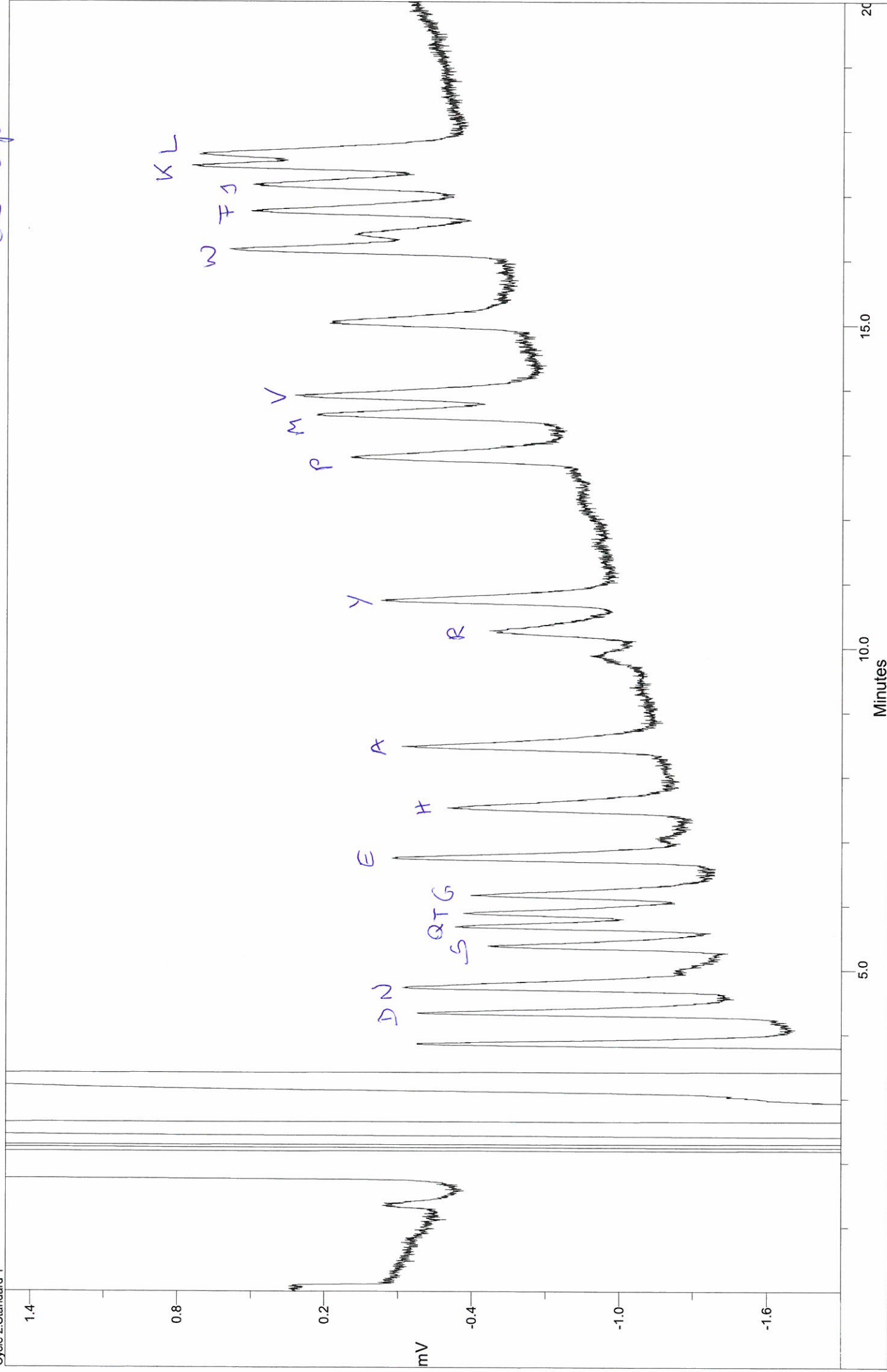

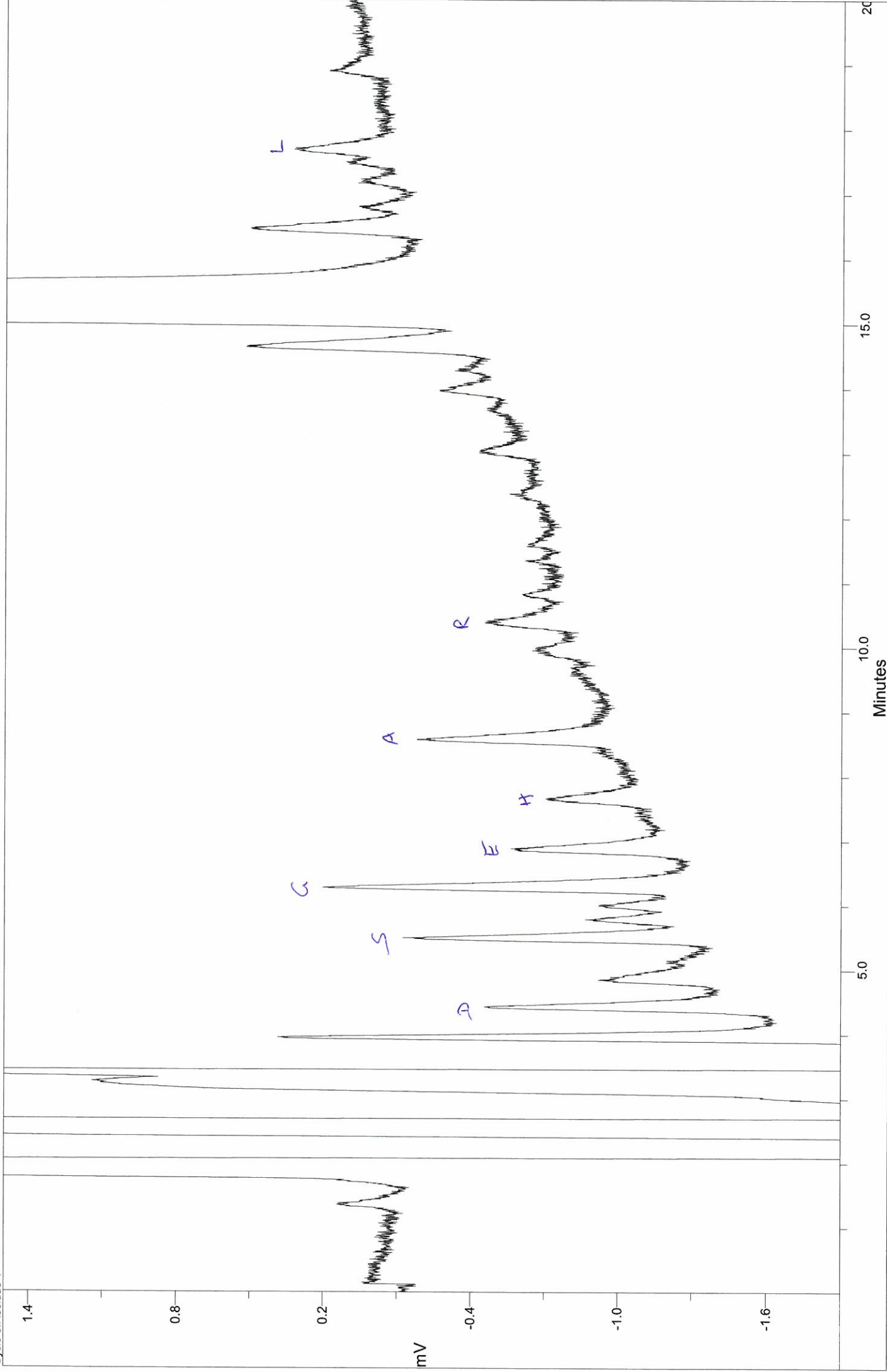

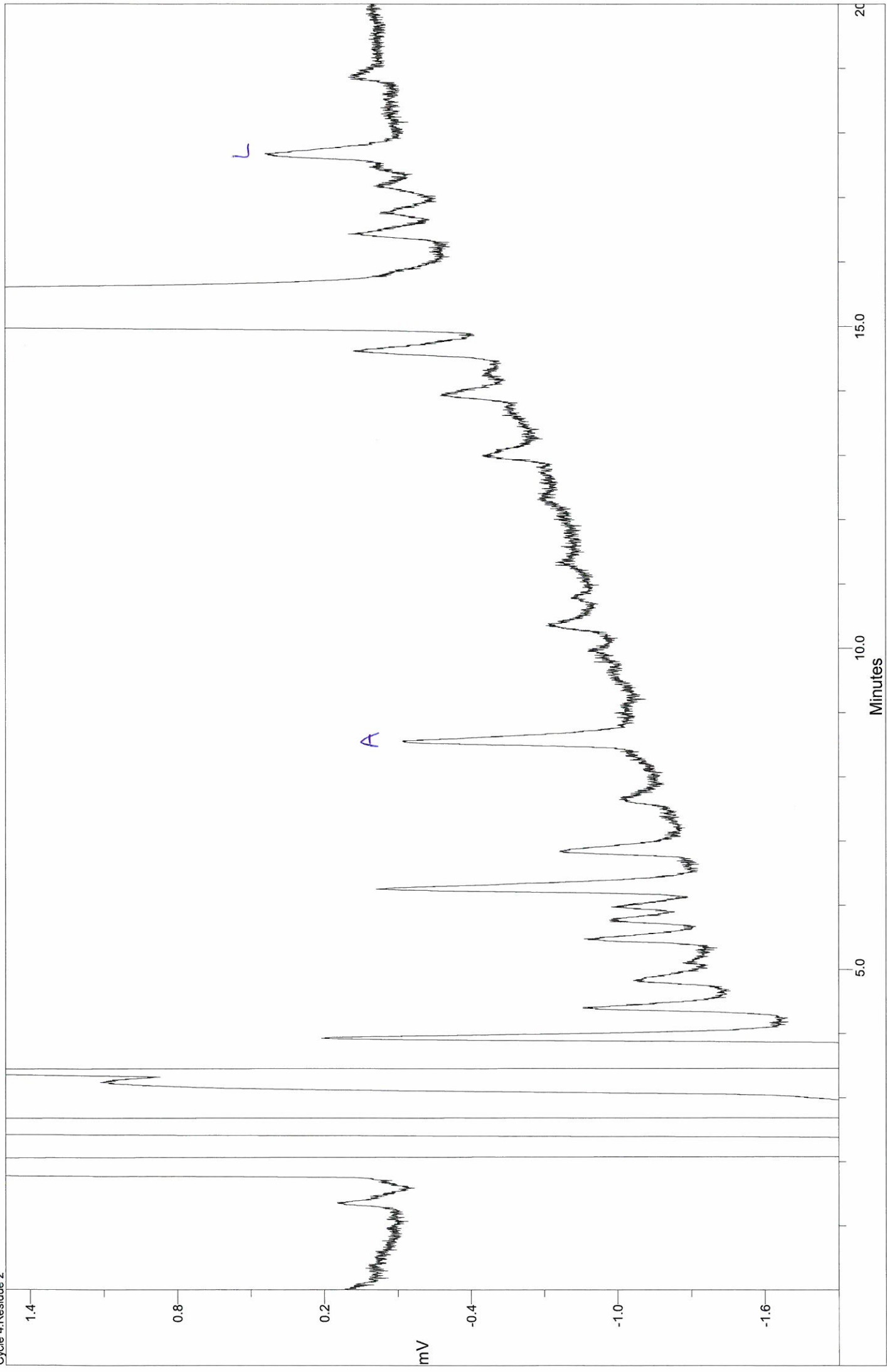

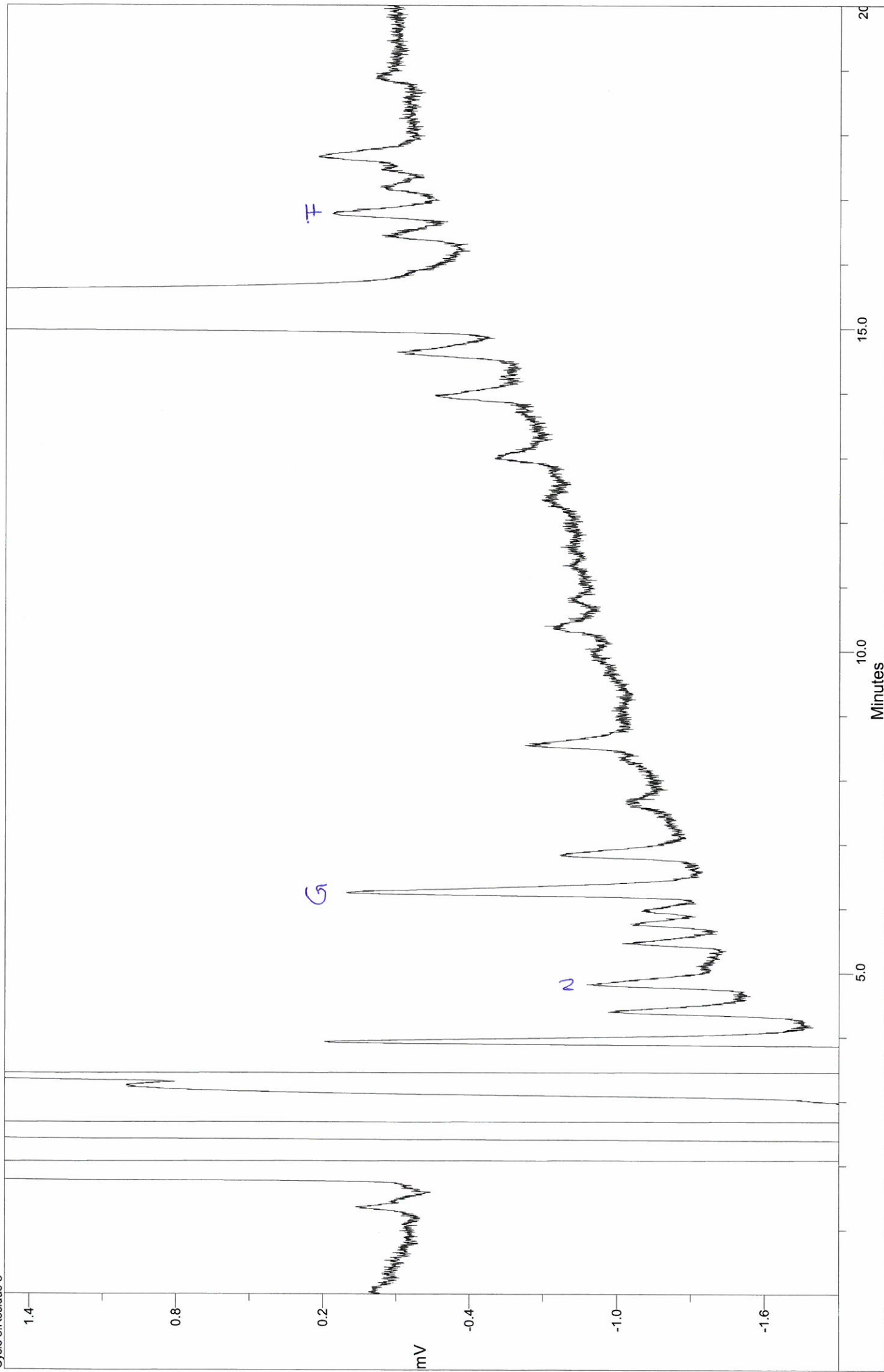

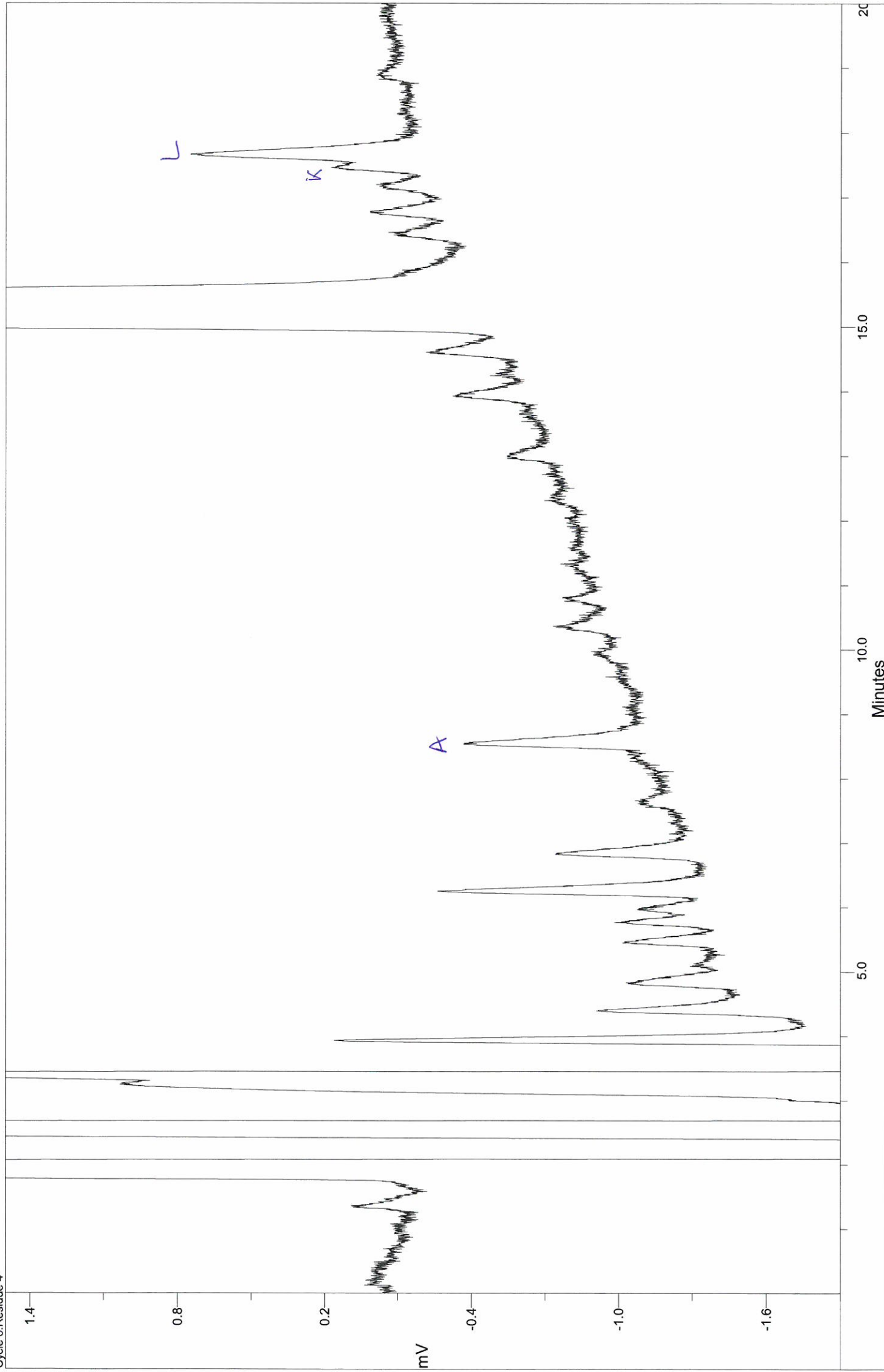

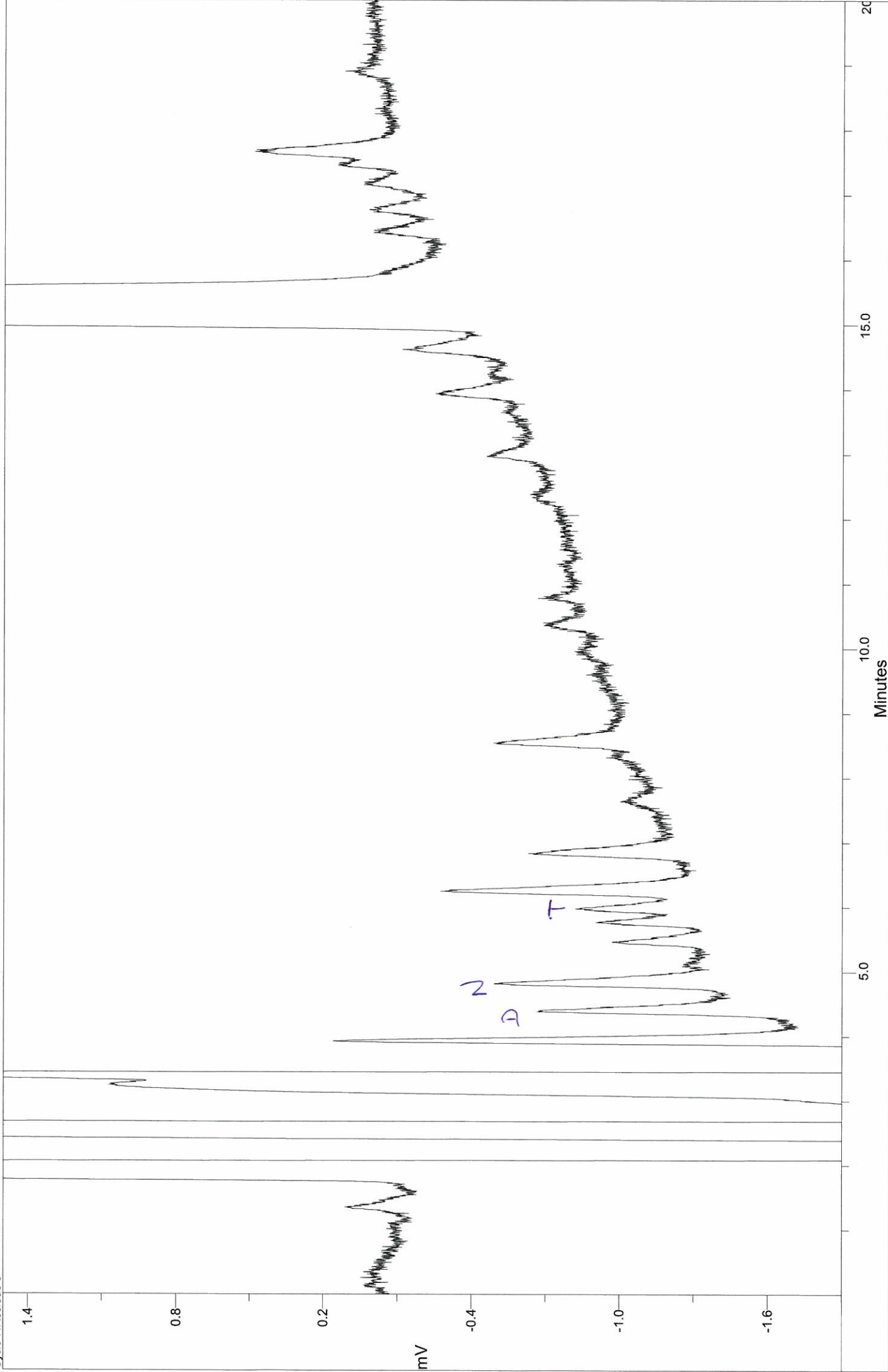

Supplement: Supplementary file 6 — 10.1186/s40064-016-2893-y Raw data of N-terminal sequence analysis of secreted Nat-Csn. [file 40064_2016_2893_MOESM6_ESM.pdf]
