# Supplementary material for: OmpA signal peptide leads to heterogenous secretion of B. subtilis chitosanase enzyme from E. coli expression system
Source: Springerplus. 2016 Jul 28;5(1):1200. doi: 10.1186/s40064-016-2893-y (PMC4963352; doi:10.1186/s40064-016-2893-y)
Supplement: Supplementary file 5 — 10.1186/s40064-016-2893-y Raw data of N-terminal sequence analysis of secreted Nat-Csn. [file 40064_2016_2893_MOESM5_ESM.pdf]

A. Univ.-Prof. Dr. Herbert Lindner
Sektion für Klinische Biochemie-Biozentrum
PROTEINSEQUENZIERUNG
Innrain 80-82
A-6020 Innsbruck

Tel.: (0512)-9003-70310
Fax : (0512)-9003-73300
herbert.lindner@i-med.ac.at

Analysenergebnis

Sequencer: ABI-Procise 492						*LAUF-Nr.* 13014		
PROBE : Csn_OmpA B 17.5.13						*Datum* 21.5.13		
ID Dietmar_Haltrich_150513						*Tel.*		
Peptid ☐	Protein ■	PVDF ■	GF ☐	BIOPR ☐	BLOT ■	Menge pM	MW 34kDa	I.Y. pM R.Y %

AS	Haupt- Sequenz	Neben- Sequenz	AS	Haupt- Sequenz	Neben Sequenz	AS	Haupt- Sequenz	Neben-. Sequenz
1	**S G A**	*DEHR*	16			31		
2	**A L G**		17			32		
3	**G N F**		18			33		
4	**L K A**		19			34		
5	**N D T**		20			35		
6			21			36		
7			22			37		
8			23			38		
9			24			39		
10			25			40		
11			26			41		
12			27			42		
13			28			43		
14			29			44		
15			30			45		

Kommentar Sequence no. 1: SAGLN starting from position 22.
Sequence no. 2: GLNKD starting from position 24.
Sequence no. 3: AGFAT starting from position 13.
N-Terminus of sample is not homogeneous!

OP BS

Dr. H. LINDNER
