# Supplementary material for: OmpA signal peptide leads to heterogenous secretion of B. subtilis chitosanase enzyme from E. coli expression system
Source: Springerplus. 2016 Jul 28;5(1):1200. doi: 10.1186/s40064-016-2893-y (PMC4963352; doi:10.1186/s40064-016-2893-y)
Supplement: Supplementary file 7 — 10.1186/s40064-016-2893-y Map of pMY202 expression vector. The pMY202 vector was used for the expression of the Bacillus chitosanase gene. This plasmid contained the E. coli OmpA signal peptide (blue) upstream from the multiple cloning site (MCS). Protein expression was under the control of a tac promoter. The vector contained an ampicillin resistance gene and a C-terminal 10×-histidine tag (red) to facilitate affinity purification. The plasmid used an origin of replication (ori) from pBR322, which has a low-copy number (15–20 plasmids per cell). [file 40064_2016_2893_MOESM7_ESM.pdf]

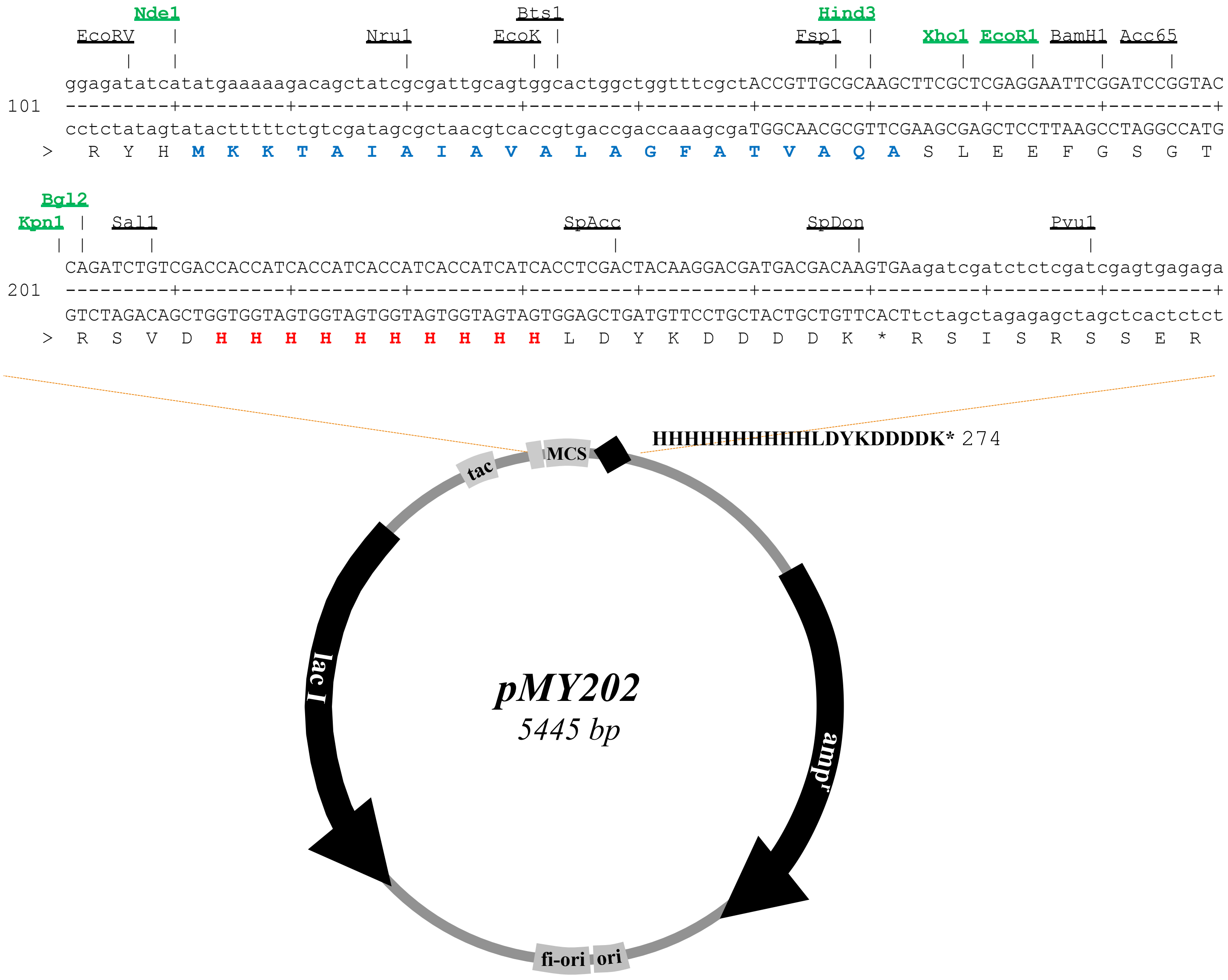

Supplementary material 1
